# Cognitive, psychosocial, and behaviour gains at age 31 years from the Jamaica early childhood stimulation trial

**DOI:** 10.1111/jcpp.13499

**Published:** 2021-08-17

**Authors:** Susan P. Walker, Susan M. Chang, Amika S. Wright, Rodrigo Pinto, James J. Heckman, Sally M. Grantham‐McGregor

**Affiliations:** ^1^ Caribbean Institute for Health Research The University of the West Indies Kingston Jamaica; ^2^ Department of Economics The University of California at Los Angeles Los Angeles CA USA; ^3^ Centre for for the Economics of Human Development University of Chicago Chicago IL USA; ^4^ American Bar Foundation Chicago IL USA; ^5^ Institute of Child Health University College London London UK

**Keywords:** Early childhood, stimulation, stunting, cognition, psychosocial skills

## Abstract

**Background:**

There is little evidence on adult benefits from early childhood interventions in low and middle‐income countries. We assessed adult cognition, psychosocial skills and behaviour from a stimulation trial conducted in Jamaica.

**Methods:**

Children with stunted growth (height‐for age <−2*SD* of references) aged 9–24 months were enrolled in a two‐year randomised‐controlled trial of nutritional supplementation and/or stimulation. At mean age 31.79 (*SD* 0.40) years, 95 of 127 participants (74.8%; 53.7% male) were assessed. Children without stunted growth were also followed as a comparison group (64 of 84 participants, 76.2%). Measurements included IQ, executive function, mental health, psychosocial skills, personality traits and risk behaviours. A block permutation test, valid for small sample sizes, was used. Analyses accounted for the randomisation protocol, multiple hypothesis testing and attrition.

**Results:**

Treatment group participants (stimulation intervention with or without supplementation, *n* = 48) had significantly greater IQ (Hedges g effect size 0. 57; 95%CI 0.20, 0.95) and cognitive flexibility (0.61; 0.25, 0.98) compared with no‐treatment (no‐intervention and supplementation only, *n* = 47). They also had reduced depressive symptoms (0.61; 0.28, 1.00), increased grit (0.53; 0.16, 0.92) and conscientiousness (0.66; 0.31, 1.07), lower substance use (rank mean score, 0.45; 0.08, 0.81) and risk taking related to health and work (0.64; 0.27, 1.00). There were 18 significant outcomes of 33 assessed. Comparison participants had higher IQ than no‐treatment (1.17; 0.81, 1.54) and treatment groups (0.62; 0.18, 1.07); and better executive function, lower social inhibition and risk taking than the no‐treatment group.

**Conclusions:**

The wide‐ranging benefits at 31 years from the stimulation intervention supports investment in larger scale programmes to promote early childhood development in disadvantaged children. The lower IQ in the treatment group compared with comparison participants, emphasises the need for continued efforts to prevent early childhood growth retardation.

## Introduction

There is increasing global attention to investment during early childhood as a strategy to reduce inequality (Daelmans et al., 2017; Richter et al., 2017). Approximately 250 million children under 5 years of age in low‐ and middle‐income countries (LMIC) do not attain their developmental potential due to poverty, stunting and inadequate stimulation (Black et al., 2017; Walker, Wachs, et al., 2011), and early childhood development programmes benefit these children’s development (Britto et al., 2017; Engle et al., 2011; Walker, Wachs, et al., 2011). Evidence, predominantly from efficacy trials in the United States, shows intensive early childhood interventions benefit adult education, behaviour, health and earnings, and reduce crime (Campbell et al., 2014; Campbell, Ramey, Pungello, Sparling, & Miller‐Johnson, 2002; Heckman & Kautz, 2014; Heckman, Moon, Pinto, Savelyev, & Yavitz, 2010b; McCormick et al., 2006). There is limited evidence of adult gains from interventions in LMIC, where resources are scarce and need greatest.

The Jamaican supplementation and stimulation study (Grantham‐McGregor, Powell, Walker, & Himes, 1991) demonstrated substantial benefits for the development of children with stunted growth. At age 22 years, stimulation benefited IQ, educational achievement, mental health and income, and reduced violent behaviour (Gertler et al., 2014; Walker, Chang, Vera‐Hernandez, & Grantham‐McGregor, 2011). The study is one of very few from LMICs to assess long‐term benefits (Tanner, Candland, & Whitney, 2015).

Our objectives were to determine whether benefits to IQ, mental health and violent behaviour continued at age 31 years, when adult outcomes may be more established, and to examine executive function, psychosocial skills, personality and risk behaviours important for adult success and well‐being (Borghans, Duckworth, Heckman, & ter Weel, 2008; Duckworth, Peterson, Matthews, & Kelly, 2007). This is part of a larger assessment including labour outcomes and physical health that will be reported separately.

## Methods

### Initial trial

The trial evaluated the separate and combined benefits of nutritional supplementation and psychosocial stimulation for development of children with stunted growth (height‐for‐age < −2 *SD* of the NCHS references: Hamill, Drizd, Johnson, Reed, & Roche, 1977) aged 9 to 24 months, identified by survey of poor neighbourhoods in Kingston, Jamaica. Homes of children with stunted growth were usually poorer than others in the neighbourhoods and typically crowded, with few possessions, outdoor sanitation and water.

Children were stratified by age (≤16 months or >16 months) and gender. The order of group assignment was determined randomly and children in each of the four strata were assigned to: supplementation only, stimulation only, both interventions or no‐intervention. Participants were enrolled by a researcher unaware of the assignment protocol, which was done by another researcher.

Children without stunted growth (height‐for‐age > −1*SD*) from the same neighbourhoods were enrolled as a comparison group. Recruitment was conducted in January–November 1987 and interventions were provided for 2 years. All groups received weekly home visits from community health workers (CHW) and free health care. CHWs had at least complete primary education and had worked in the primary health system. CHWs who delivered stimulation were given 4 weeks training.

The supplement comprised 1 kg of milk‐based formula delivered weekly. The stimulation intervention aimed to increase mothers’ ability to promote development through play and mother‐child interaction. At the visits, CHWs conducted a play session with mother and child to demonstrate activities. Emphasis was placed on encouraging mothers’ responsiveness, use of language and praise, and integration of activities into daily routines. Homemade play materials and simple picture books were provided and exchanged each week. Supervisors monitored visit quality through monthly observations with each CHW and met them individually every week to discuss their visits and resolve difficulties. This ensured few visits were missed. For further details on trial design and interventions, see Grantham‐McGregor et al., (1991) and Grantham‐McGregor and Smith (2016). Trial registration ISRCTN 31299262.

**Figure 1:**
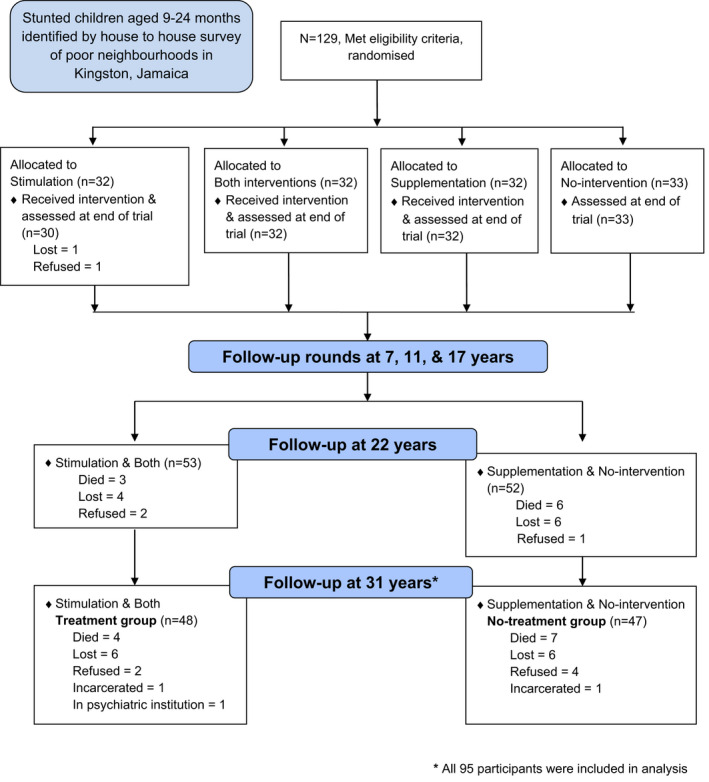
Profile of trial and follow‐up

Of 129 children with stunted growth enrolled, 127 completed the trial: no‐intervention 33, supplementation 32, stimulation 30, supplementation and stimulation 32. Participants were re‐surveyed at ages 7, 11, 17 and 22 years (Figure 1 and Appendix S1). At 7 years, 52 children without stunted growth, identified in the initial survey, were added to the original 32 comparison children. At age 22, 105 of the 127 participants with stunted childhood growth were located (Walker, Wachs, et al., 2011).

### Measurements at age 31 years

#### IQ

Cognitive skills were measured using the four subtests of the Wechsler Abbreviated Scale of Intelligence (WASI‐II), giving three composite scores: Perceptual reasoning, Verbal comprehension and Full‐scale IQ (Wechsler, 2011).

#### Executive function

Verbal fluency with category switching, Card sorting, Colour–word interference and Tower test were selected from the Delis‐Kaplan Executive Function System. These measured fluent productivity, cognitive flexibility, inhibitory control and planning and reasoning (Delis, Kaplan, & Kramer, 2001). Due to concerns during data collection, we examined the Colour‐Word test scores by participant reading ability. Participants with reading below grade 3.0 (26% of participants) had better scores suggesting that low reading ability reduced colour‐word interference. We therefore omitted this test.

#### Mental health

Depressive symptoms, anxiety and social inhibition were measured using scales used previously – the CES‐Depression scale (Radloff, 1977), State Trait Anxiety Inventory (Spielberger, Gorsuch, Lushene, Vagg, & Jacobs, 1983) and social inhibition from the Inventory on Interpersonal Problems (Horowitz, Alden, Wiggins, & Pincus, 2000).

#### Psychosocial skills

Grit was measured using the Duckworth Scale (Duckworth & Quinn, 2009), self‐esteem using the Rosenberg Scale (Rosenberg, 1965) and self‐control with items from the Brief Self‐Control questionnaire (Tangney, Baumeister, & Boone, 2004).

#### Personality Traits

The Big Five personality traits were measured with the ten item Personality Inventory (Gosling, Rentfrow, & Swann, 2003).

#### Substance use

Drug and alcohol use was measured with the WHO ASSIST questionnaire (Ali et al., 2002).

#### Violent behaviour

A questionnaire was used similar to age 22. Factor analysis with principal component analysis and varimax rotation was conducted to reduce the number of variables and identify underlying constructs. This yielded two factors with eigen value >1 and regression scores were computed. Factor 1 ‘fights and weapons’, included involvement in fights and use of weapons other than guns; factor 2 ‘guns and gangs’ included threatening someone with a gun, shooting a gun and gang membership.

#### Risk taking

This was assessed with five risk attitude questions from Dohmen et al. (2005), each with scores ranging from 1 to 5. Factor analysis (as described above) yielded 2 factors with eigen value >1, one comprising general and financial risks and another comprising risks related to health, work and trust in others. The average score of the variables on each factor was used.

Scales not used previously in Jamaica were piloted, and test‐retest reliability determined (no. of respondents = 19). Intra‐class correlation coefficients (ICC) were ≥0.88. For executive function tests (*n* = 8), ICC for Verbal Fluency category switching was 0.55, Card Sorting total correct 0.71 and Tower score 0.56.

Inter‐observer reliability was determined for all measurements, ICC coefficients were ≥ 0.95 for all scales and tests. Internal validity (cronbach’s alpha) was 0.71 for social inhibition, 0.59 for grit, 0.53 for self‐control and ≥ 0.83 for anxiety, depression and self‐esteem.

### Procedure

Participants were contacted using previous locator information. Following informed consent, participants residing in Jamaica were assessed at our research unit. IQ and executive function tests were administered followed by questionnaires. We attempted to relocate migrants measured at age 22 and participants who migrated subsequently. Migrants lived in the United Kingdom, the United States and Canada. The majority were measured in their homes and a few at locations convenient to them. Measurements were conducted by three persons, blind to participants’ group assignment, between May and December 2017.

### Ethics

Ethical approval was obtained from the University of the West Indies Ethics Committee. Written informed consent was obtained. US $50 or equivalent was given to each participant. Assessments were conducted in private and participants identified by ID number only on all tests/questionnaires.

### Analysis

The trial used stratified randomisation based on child age on enrolment ≤16 months or >16 months and gender. These variables, as well as whether child received supplementation, and whether the mother had secondary education (which differed between treatment and no‐treatment groups) are used to partition the data into subsets that contain both treatment and no‐treatment participants (Appendix S2). The estimation is based on linear regression, conditioned on binary indicator variables for each subset. Our treatment effect estimator is a weighted average of the treatment effects across strata and can be interpreted as a non‐parametric ANOVA estimation (see Appendix S3). Our inference is based on a non‐parametric block‐permutation test that explores an exchangeability property arising from the randomisation protocol, that under the null hypothesis the outcome distribution of the treatment and no‐treatment groups are equal. Permutation tests do not rely on the asymptotic behaviour of test statistics of the targeted population. Instead, permutation tests use the exact small sample distribution of a test statistic. The distribution consists of all the values of the test statistic generated by swapping the treatment status among participants that belong to the same randomisation stratum. The method accounts for the complexity of the randomisation and secures robust inference in small sample sizes (for further information, see S2–S3 and Gertler et al., 2014; Heckman et al., 2010b; Heckman, Moon, Pinto, Savelyev, & Yavitz, 2010a; Heckman, Pinto, & Savelyev, 2013). We seek to reject the null hypothesis that the no‐treatment group outperforms the treatment group in favour of the alternative hypothesis that the treated participants outperform the no‐treatment participants.

We account for multiple outcomes using the stepdown procedure (Romano & Wolf, 2005) that controls for family‐wise error rate. Outcomes are grouped by assessment type (e.g. executive function) to secure interpretation of the joint hypothesis of no treatment effect across multiple outcomes. We also compute the rank‐average of each participant across the outcome groups. This variable can be understood as a non‐parametric index that aggregates performance across multiple outcomes.

Following previous literature, we examined treatment effects by gender (Elango, Garcia, Heckman, & Hojman, 2015). To determine whether migration influenced the overall findings, we also evaluate the subset of non‐migrants only. We correct for potential bias generated by non‐random attrition using augmented inverse probability weighting (AIPW), which improves the standard IPW by using the control variables to forecast the outcome and estimate the attrition probability The results are qualitatively the same as those presented in the main text. We also compare treatment and no‐treatment participants with the comparison group. This analysis is not causal as the comparison children were not part of the randomisation and participants with stunted growth and those without differ not only on observed variables in the data but also unobserved variables that can affect the outcomes.

## Results

Ninety‐five of the 127 participants who completed the trial were measured (74.8%) and 64 of 84 comparison participants (76.2%). Attrition in the trial was 27.7% in the no‐treatment group and 22.6% in the treatment group. Reasons for loss are shown in Figure 1.

The only differences in enrolment characteristics were child weight‐for‐height and mother’s education (Table 1). There were no differences between the groups at follow‐up in age, proportion of migrants and housing quality. Table S1 shows that conditional analyses using block‐permutation inference eliminates the baseline differences.

**Table 1:** Participant characteristics on enrolment and follow‐up (no‐treatment group *n* = 47, treatment group *n* = 48)

	No‐treatment (*n* = 47)		Treatment (*n* = 48)		Group difference	Effect size^c^	*t*‐Statistic	*p*‐Value^d^
	Mean	*SD*	Mean	*SD*				
*Child characteristics on enrolment*								
Age in months	19.13	4.30	19.01	4.15	−0.12	−0.03	−0.14	.893
Male	0.55	0.50	0.52	0.50	−0.03	‐0.06	−0.31	.755
Birth weight	2.95	0.46	2.81	0.50	−0.14	−0.31	−1.44	.154
*Z*‐score (Weight for height)	−0.89	0.71	−1.19	0.71	−0.30	−0.42	−2.05	.043
*Z*‐score (Height for age)	−2.87	0.62	−2.98	0.58	−0.11	−0.17	−0.86	.390
Developmental quotient	97.06	9.30	99.02	8.83	1.96	0.21	1.05	.295
Housing score	7.51	1.83	7.23	1.45	−0.28	−0.15	−0.83	.407
*Parent characteristics on enrolment*								
HOME^a^ score	17.06	4.91	15.81	3.84	−1.25	−0.25	−1.39	.169
Mothers PPVT^b^	85.36	17.95	85.52	21.56	0.16	0.01	0.04	.969
Young mother (age <20 years) indicator	0.23	0.43	0.25	0.44	0.02	0.04	0.18	.858
Mother education (secondary exams) indicator	0.23	0.43	0.08	0.28	−0.15	−0.35	−2.04	.044
Mother working indicator	0.17	0.38	0.25	0.44	0.08	0.21	0.95	.345
*Follow‐up characteristics*								
Participant age (years)	31.79	0.39	31.80	0.41	0.01	0.02	0.07	.942
Migrant indicator	0.17	0.38	0.17	0.38	−0.00	−0.01	−0.05	.964
Housing (factor score)	−0.08	1.07	0.04	0.89	0.12	0.11	0.59	.557

Birth weight, housing score at follow‐up treated group *n* = 47.

^a^ Home observation for measurement of the environment (Caldwell & Bradley, 1984).

^b^ Peabody picture vocabulary test (Dunn & Dunn, 2007).

^c^ Hedges *g* effect size (Rosnow & Rosenthal, 2003).

^d^ Two‐sided *p*‐value for difference between treatment and no‐treatment means.

### Treatment effects

Table 2 presents treatment effects for IQ, executive functioning, mental health, psychosocial skills and personality traits. Table 3 shows effects for substance use, risk taking and violent behaviour. Hedges g effect sizes (Rosnow & Rosenthal, 2003) are presented in the tables.

**Table 2:** Treatment effects on cognitive, mental health, psychosocial and personality outcomes

	No‐treatment (*n* = 47)		Treatment (*n* = 48)		Treatment effect^b^	Effect size^c^ (95% CI)	Asymptotic (1‐sided)		Permutation (1‐sided)	
	Mean	*SD*	Mean	*SD*			*t*‐Statistic	*p*‐Value^d^	Single *p*‐value^e^	Stepdown^f^
*WASI‐II IQ scores*										
Full scale IQ	72.19	10.47	78.17	11.83	5.98	0.57 (0.20,0.95)	2.42	.009	.006	.012
Perceptual reasoning	72.12	11.15	78.38	12.59	6.25	0.56 (0.19,0.94)	2.38	. 010	.007	.013
Verbal comprehension	76.85	11.89	81.75	12.70	4.91	0.41 (0.04,0.80)	1.81	.037	.035	.035
Rank mean	0.44	0.23	0.56	0.27	0.11	0.49 (0.11,0.86)	2.02	.023	.015	–
*Executive function*										
Fluency accuracy	8.17	2.77	8.75	3.16	0.58	0.21 (−0.17,0.61)	0.89	.188	.176	.176
Fluency:% switching accuracy	8.55	3.30	9.51	3.06	0.96	0.29 (−0.06,0.68)	1.36	.088	.087	.195
Card sorting total score	4.40	2.74	6.08	2.88	1.68	0.61 (0.25,0.98)	2.70	.004	.004	.015
Card sort description	4.47	2.61	5.95	2.89	1.48	0.56 (0.20,0.93)	2.43	.009	.007	.025
Tower achievement score	8.47	3.23	9.29	1.99	0.81	0.25 (−0.11,0.64)	1.38	.086	.131	.225
Rank mean	0.45	0.20	0.55	0.19	0.10	0.50 (0.11,0.87)	2.32	.011	.010	–
*Mental Health*										
Depressive symptoms^a^	−20.50	9.07	−14.98	9.96	5.52	0.61 (0.28,1.00)	2.62	.005	.001	.003
Anxiety^a^	−43.22	8.22	−40.43	6.62	2.79	0.34 (−0.01,0.70)	1.69	.047	.054	.054
Social inhibition^a^	−6.60	3.31	−5.39	3.27	1.21	0.36 (−0.01,0.75)	1.67	.049	.051	.095
Rank mean	0.43	0.22	0.56	0.20	0.13	0.60 (0.24,0.96)	2.82	.003	.003	
*Psychosocial Skills*										
Self esteem	21.58	5.16	22.74	4.34	1.16	0.22 (−0.13,0.59)	1.10	.137	.151	.151
Grit	24.39	4.36	26.72	3.74	2.32	0.53 (0.16,0.92)	2.59	.006	.010	.027
Self‐control	9.08	3.29	9.92	2.92	0.83	0.25 (−0.11,0.63)	1.21	.115	.124	.221
Rank mean	0.45	0.22	0.55	0.19	0.10	0.44 (0.07,0.81)	2.11	.019	.027	–
*Personality traits*										
Extraversion	8.39	2.44	8.55	2.59	0.16	0.07 (−0.39,0.43)	0.29	.388	.383	.581
Agreeableness	11.67	2.30	11.83	2.48	0.16	0.07 (−0.29,0.47)	0.30	.383	.380	.735
Conscientious	11.14	2.71	12.96	1.87	1.82	0.66 (0.31,1.07)	3.54	.001	.001	.001
Emotional stability	9.94	2.72	10.22	2.34	0.28	0.10 (−0.25,0.47)	0.50	.311	.324	.736
Open to experiences	10.47	2.25	10.52	2.70	0.04	0.02 (−0.32,0.39)	0.08	.468	.459	.459
Rank mean	0.47	0.15	0.53	0.15	0.06	0.41 (0.05,0.79)	1.84	.035	.037	–

Estimates are based on a block permutation inference conditional on main variables at the onset of the intervention.

^a^ Variable is reversed (multiplied by −1) to report treatment effects consistently in positive direction.

^b^ Estimated difference in means between groups.

^c^ Hedges *g* effect size Rosnow and Rosenthal (2003).

^d^ Single hypothesis testing of difference between treatment groups.

^e^ Mid‐*p*‐value based on 15.000 permutations draws, using pre‐pivoted treatment effect estimate and permutation scheme is either na¨ıve or block permutation.

^f^ Multiple hypothesis testing (stepdown).

**Table 3:** Treatment effects on Risk Behaviours and Violence

	No‐treatment (*n* = 47)		Treatment (*n* = 48)		Treatment effects^b^	Effect size^c^ (95% CI)	Asymptotic (1‐sided)		Permutation (1‐sided)	
	Mean	*SD*	Mean	*SD*			*t*‐Statistic	*p*‐Value^d^	Single *p*‐value^e^	Stepdown^f^
*Substance abuse (WHO‐ASSIST)*										
Aggregate alcohol score^a^	−3.58	2.71	−2.52	3.22	1.06	0.39 (0.05,0.84)	1.61	.055	.026	.047
Aggregate marijuana score^a^	−3.23	3.69	−2.03	3.74	1.20	0.32 (−0.02,0.75)	1.46	.074	.064	.071
Rank mean	0.46	0.19	0.54	0.21	0.09	0.45 (0.08, 0.81)	1.97	.026	.019	–
*Risk taking scores*										
General risk & finance^a^	−3.92	0.77	−3.68	1.12	0.24	0.31 (−0.07,0.65)	1.13	.131	.081	.081
Health, work & trust^a^	−3.25	0.92	−2.66	0.93	0.59	0.64 (0.27,1.00)	2.89	.002	.002	.004
Rank mean	0.45	0.18	0.55	0.24	0.10	0.57 (0.21,0.94)	2.20	.015	.008	–
*Violence factor scores*										
Fights & weapons^a^	−0.10	1.01	0.23	0.50	0.33	0.33 (0.01,0.93)	1.89	.031	.047	.093
Guns & gangs^a^	0.30	0.49	0.18	0.55	−0.12	−0.24 (−0.54,0.46)	−1.02	.844	.732	.732
Rank mean	0.51	0.05	0.49	0.11	−0.01	−0.26 (−1.20,0.05)	−0.72	.764	.778	–

Estimates are based on a block permutation inference conditional on main variables at the onset of the intervention.

^a^ Variable is reversed (multiplied by −1) to report treatment effects consistently in positive direction.

^b^ Estimated difference in means between groups.

^c^ Hedges *g* effect size Rosnow and Rosenthal (2003).

^d^ Single hypothesis testing of difference between treatment groups.

^e^ Mid‐*p*‐value based on 15.000 permutations draws, using pre‐pivoted treatment effect estimate and permutation scheme is either na¨ıve or block permutation.

^f^ Multiple hypothesis testing (stepdown).

#### IQ

The treatment group had significant gains in full scale IQ scores, verbal and perceptual sub‐scales with moderate effect sizes.

#### Executive functioning

Significant gains in the card sort scores indicate greater cognitive flexibility. The rank mean score indicates an overall gain for executive function tests.

#### Mental health

The treatment group had reduced depressive symptoms. Anxiety, and social inhibition were not significantly different. Rank mean analysis showed overall better mental health.

#### Psychosocial skills

Treated participants had higher grit scores. Self‐esteem and self‐control were not significantly different. Rank mean analysis showed an overall effect.

#### Personality traits

The treatment group had significantly higher conscientiousness. There were no other differences in personality.

#### Substance use

The treatment group had reduced alcohol use but no significant difference in marijuana use, the rank mean score indicates reduced overall substance use.

#### Risk taking

The treatment group were less likely to take risks related to health, work and trust in others. Rank scores also indicate reduced risk taking.

#### Violent behaviour

There were no significant differences in fights and use of weapons, gun use and gang membership, or in overall rank scores.

#### Comparisons for non‐migrants

The pattern of treatment effects for non‐migrants was similar to the total sample (Tables S2 and S3).

#### Comparisons of males and females

Exploratory analyses by gender showed no differences in treatment effects other than reduced risk taking in women only (Tables S2 and S3).

Correction for attrition produced qualitatively equivalent results (Tables S4–S7).

#### Longitudinal findings

**Figure 2:**
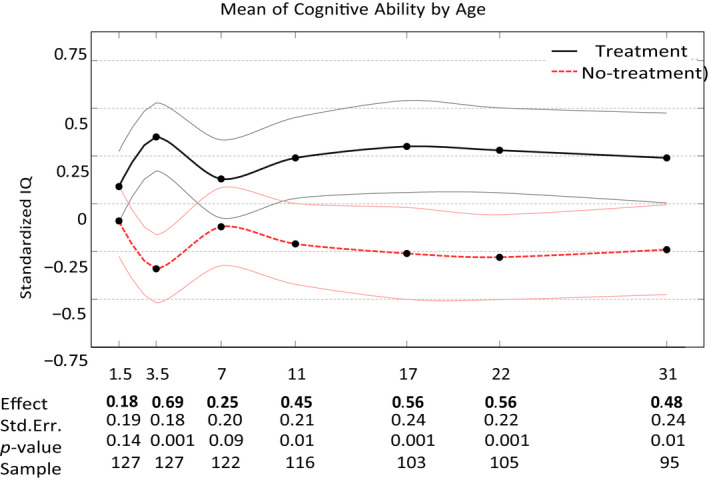
Evolution of Standardized IQ Measures by Age of Participants of the Jamaican Study. This figure presents the mean of the cognitive measures for the Jamaican study participants by age. Data consists of seven variables: the standardized Griffiths developmental quotient measured at mean age 18 and 42 months old; the standardized Stanford Binet IQ surveyed at age 7 years; the standardized WISC Full Scale full scale IQ at age 11; the standardized WAIS Full scale IQ measured at ages 17, 22 and the standardized WASI full scale at 31. The thick solid line presents the conditional estimates for the mean of the cognitive measures for stimulation arms of the intervention controlled by age variation. The thick dashed line presents the respective estimates for the non‐stimulation arms of the intervention. The boundaries denote estimated standard errors of each mean. The bottom of the figure displays four rows. The first one presents the treatment effect estimate controlled for age variation. The second row presents the estimated standard error. The third row presents the one‐sided single hypothesis *p*‐value associated with the null hypothesis of no treatment effect. The last row presents the sample size

Figure 2 compares developmental quotients or IQ in standardised scores from enrolment through to age 31. The treatment effect at the end of the trial was 0.69*SD*. No significant difference in IQ was detected at 7 years, however, the impact increased at age 11 years and was also significant at 17, 22 and 31 years. The treatment group had significantly reduced depressive symptoms at 17, and this remained lower at 22 and 31 years.

### Analysis with comparison group

Differences between the comparison group and the no‐treatment and treatment groups were examined (Tables S8–S11). Comparison participants had higher IQ than the no‐treatment and treatment groups. They also had better scores in executive function, lower social inhibition and risk taking compared with the no‐treatment group only. The comparison group had higher levels of violent behaviour than the treatment and no‐treatment groups.

## Discussion

Participants from the Jamaica stimulation trial were followed to age 31 years and showed sustained intervention benefits to IQ and depressive symptoms. Benefits to executive function, grit and conscientiousness, lower substance use and risk taking are shown for the first time. This is the longest follow up of a randomised controlled trial of an early childhood stimulation intervention in LMIC and included a wide range of measurements. Retention was reasonable for follow‐up of this duration.

The initial impact of the intervention on cognition declined and was no longer significant at age 7 years (Grantham‐McGregor, Walker, Chang, & Powell, 1997), however, significant IQ benefits have been evident consistently from age 11 through to this follow‐up when effects were substantial (0.57). In contrast, in US studies IQ benefits generally faded after a few years (Elango et al., 2015) although they remained in the intensive Abcedarian study (Campbell et al., 2012).

Improvement in mental health with lower depressive symptoms is a robust finding, present since age 17 (Walker, Chang, Powell, Simonoff, & Grantham‐McGregor, 2006; Walker, Wachs, et al., 2011). Depression is associated with lower job performance, social functioning and child‐care (Adler et al., 2006; Kupferberg, Bicks, & Hasler, 2016). Benefits to depression have been found in some US studies (Palfrey et al., 2005; Reynolds, Temple, Ou, Artega, & White, 2011) but not others (Campbell et al., 2012). Reductions in violent behaviour observed at age 22 were no longer evident and the earlier age may be a higher risk period for violence in this population.

We found gains in cognitive flexibility which is the ability to change perspectives and adjust to new demands. Cognitive flexibility is related to reasoning, planning and creativity, attributes likely to contribute to job performance (Diamond, 2013). Treatment increased conscientiousness, the personality trait most consistently related to adult success (Borghans et al., 2008; Heckman & Kautz, 2014), job performance (Barrick & Mount, 1991; Borghans et al., 2008; Heckman & Kautz, 2014) and wealth (Duckworth, Weir, Tsukayama, & Kwok, 2012). Benefits to executive function and positive personality were reported in Perry Preschool participants (Heckman & Karapakula, 2019).

The treated group also had higher levels of grit. Most literature on grit is from high income countries and questions whether grit is a separate trait or part of conscientiousness as grit correlated highly with conscientiousness and moderately with emotional stability (Credé, Tynan, & Harms, 2017). However, in our disadvantaged sample, correlation between grit and conscientiousness was low (*r* = .25) and similar to that with emotional stability (*r* = .26). One adult follow‐up that measured grit found no benefits (Heckman & Karapakula, 2019).

Alcohol and overall substance use were lower in the treated group. Reduced marijuana use (Campbell et al., 2002) and reduced drug and alcohol abuse (Reynolds et al., 2011), have been reported in US studies. No difference in alcohol and marijuana use has also been reported (Muennig, Schweinhart, Montie, & Neidell, 2009).

The mechanisms for sustained intervention benefits are unknown, however, the intervention aimed to improve task‐orientation and sustained attention and included many problem‐solving activities. Children’s efforts and success in completing tasks also received much positive feedback and celebration. These may have contributed to benefits in grit, executive function and possibly conscientiousness. We also focused on building the mothers’ self‐esteem and child‐rearing skills which may have contributed to sustaining benefits. Finally, the initial cognitive benefits were large and may have contributed to gains in other domains.

Small sample size is a study limitation. We used a permutation test appropriate for small sample inference. The current sample size has 80% power at 5% significance test to detect a difference of 0.5*SD*, using a one‐sided test. We adjusted for multiple comparisons within domains and significant findings were of moderate to large effect size. Nonetheless, multiple testing is still a concern given the number of outcomes and subgroup analyses. For two of the executive function tests, the test‐retest reliability was low. There were few statistically significant differences on enrolment, with lower maternal education and child weight‐for‐height in the treatment group. Mothers with less education, and their children, may respond differently to interventions and maternal education was included in the conditional variables.

The study provides evidence of life‐course benefits from a relatively low input intervention across cognitive, psychosocial and behavioural variables that may reduce intergenerational poverty transmission. The participants were extremely disadvantaged, and the level of disadvantage in untreated groups increases the likelihood of long‐term gains (Bailey, Duncan, Odgers, & Yu, 2017; Elango et al., 2015; Heckman & Mosso, 2014). Thus, the findings may not be generalisable to children with less disadvantage.

Analysis with the comparison group showed that the no‐treatment group had large deficits in IQ and cognitive flexibility. Despite substantial gains, IQ also remained lower in the treatment group. Stunting affects 149 million children under 5 years (UNICEF, 2019) and these findings reinforce the need to reduce stunting and provide affected children with interventions to support development. The intervention has been used in several countries (Grantham‐McGregor & Smith, 2016) and future work will explore mechanisms such as curriculum content, size of initial benefit, changes in parent behaviour, and later investments in children that underlie sustainability of benefits to inform scale up.

## Conclusions

This low‐cost, feasible early childhood intervention had life changing effects, similar to higher input interventions (Campbell et al., 2002; Heckman et al., 2013), and the findings support further expansion. Nonetheless, differences in IQ remained between participants with stunted growth and the comparison group.

## Supporting information


**Appendix S1.** Attrition at 31 years old.
**Appendix S2.** Sampling variation and permutation blocks.
**Appendix S3.** Description of empirical evaluations.
**Table S1.** Conditional block permutation inference on participants characteristics using *t*‐statistic – all data.
**Table S2.** Treatment effects on cognitive, psychosocial and personality outcomes for non‐migrants and by gender.
**Table S3.** Treatment effects on risk taking and violence behaviors for non‐migrants and by gender.
**Table S4.** AIPW inference on cognitive, psychosocial and personality outcomes conditional all data.
**Table S5.** AIPW inference on risk taking and violence behaviors conditional all data.
**Table S6.** AIPW inference on cognitive, psychosocial and personality outcomes conditional summary.
**Table S7.** AIPW inference on risk taking and violence behaviors conditional summary.
**Table S8.** Comparison vs. no‐treatment conditional inference on cognitive, psychosocial and personality outcomes.
**Table S9.** Comparison vs. no‐treatment conditional inference on risk taking and violence behaviors.
**Table S10.** Comparison vs. treatment conditional inference on cognitive, psychosocial and personality outcomes.
**Table S11.** Comparison vs. treatment conditional inference on risk taking and violence behaviors.
