# Supplementary material for: Cognitive, psychosocial, and behaviour gains at age 31 years from the Jamaica early childhood stimulation trial
Source: J Child Psychol Psychiatry. 2021 Aug 17;63(6):626–35. doi: 10.1111/jcpp.13499 (PMC8850528; doi:10.1111/jcpp.13499)
Supplement: Supplementary file 1 — Appendix S1. Attrition at 31 years old. Appendix S2. Sampling variation and permutation blocks. Appendix S3. Description of empirical evaluations. Table S1. Conditional block permutation inference on participants characteristics using t‐statistic – all data. Table S2. Treatment effects on cognitive, psychosocial and personality outcomes for non‐migrants and by gender. Table S3. Treatment effects on risk taking and violence behaviors for non‐migrants and by gender. Table S4. AIPW inference on cognitive, psychosocial and personality outcomes conditional all data. Table S5. AIPW inference on risk taking and violence behaviors conditional all data. Table S6. AIPW inference on cognitive, psychosocial and personality outcomes conditional summary. Table S7. AIPW inference on risk taking and violence behaviors conditional summary. Table S8. Comparison vs. no‐treatment conditional inference on cognitive, psychosocial and personality outcomes. Table S9. Comparison vs. no‐treatment conditional inference on risk taking and violence behaviors. Table S10. Comparison vs. treatment conditional inference on cognitive, psychosocial and personality outcomes. Table S11. Comparison vs. treatment conditional inference on risk taking and violence behaviors. [file JCPP-63-626-s001.docx]

**Supporting Information**

**Contents**

**Appendix S1.** Attrition at 31 Years Old

**Appendix S2.** Sampling Variation and permutation Blocks

How does a Permutation Test Work?

How to define Permutation Blocks?

The Comparison Sample

**Appendix S3.** Description of Empirical Evaluations

**Table S1-S11**

# Appendix S1. Attrition at 31 Years Old

Our final sample of analysis consists of 95 participants. Thirty-two (25%) of the 127 original participants were not interviewed at age 31. The attrition rate increased since the 22 years old follow-up. At age 22, the attrition rate was 17% and consisted of 22 individuals. Ten of these individuals were not found, 9 died, and 3 of those who were found but refused to be interviewed. Of the 13 that were not found or refused to be interviewed, 9 were migrants. [Gertler et al.](#_bookmark22) ([2014](#_bookmark22)) shows that the treatment status is not a significant predictor of the overall probability of attrition and the baseline means of none of the baseline variables are significantly different between the groups that dropped out and the group that stayed in the sample, even when we stratify by treatment and no-treatment groups. They concluded that the potential selection bias due to non-random attrition at age 22 is not of main concern. At age 31, there are 10 additional participates that were not interviewed. These participants are evenly distributed across randomization arms of the intervention. The following table describes the attrition patters across the multiple surveys of the intervention.

|  | **Treatment Arms**  **No-treatment Supplementation Stimulation Both Treatments** | sum |
| --- | --- | --- |

| **Onset** | 33 | 32 | 32 | 32 | 129 |
| --- | --- | --- | --- | --- | --- |
| Did not Complete |  |  | 2 |  | 127 |

| **7 y.o.** | **follow-up** | 32 | 31 | 29 | 30 | 122 |
| --- | --- | --- | --- | --- | --- | --- |
| **11 y.o.** | **follow-up** | 31 | 30 | 27 | 28 | 116 |
| **17 y.o.** | **follow-up** | 27 | 28 | 21 | 27 | 103 |
| **22 y.o.** | **follow-up** | 26 | 26 | 24 | 29 | 105 |

| **31 y.o. follow-up** | 23 24 22 26 | 95 |
| --- | --- | --- |

**22 y.o. follow-up**

| **Died** | 4 | 2 | 2 | 1 | 9 |
| --- | --- | --- | --- | --- | --- |
| **Refused** | 0 | 1 | 1 | 1 | 3 |
| **Lost** | 3 | 3 | 3 | 1 | 10 |

| **31 y.o. Attrition** | 3 2 2 3 | 10 |
| --- | --- | --- |

We observe that the attrition pattern continues to be reasonably balanced across the multiple surveys of the intervention. The pattern of attrition is also balanced in terms of baseline variables across treatment arms as shown in Table 1 of the main paper. We perform further analysis that accounts for the possibility of non-random attrition. We apply the method of Augmented Inverse Propensity Weighting (AIPW) of [Glynn and Quinn](#_bookmark23) ([2010a](#_bookmark23)); [Robins et al.](#_bookmark27) ([1994](#_bookmark27)). The method corroborates our expectation that non-random attrition is not a source of major concern.

# Appendix S2. Sampling Variation and Permutation Blocks

As mentioned, we were able to find and interview 95 out of the original 127 (75%) participants who completed the program. The sample remained balanced as we only observe significant differences in 2 out of 15 variables (see Table 1 of the main paper). Mothers of children in the treatment group were more likely to have completed **less** schooling than mothers of children in the no-treatment group. We use the indicator of secondary exams completion as a measurement of schooling achievement. Children in the treatment group also had **lower** weight for height than children in the no-=treatment group.

These imbalances are already present in the full baseline sample of 127, which suggests that they were the result of sampling variation in the original randomization rather than differential sample attrition. Moreover, these imbalances are more likely to reduce the treatment effects as children in the no-treatment group have mothers with slightly higher education. We control for baseline imbalances using non-parametric permutation inference that condition on the imbalanced pre-program variables using blocks of permutation. The next section describes the permutation blocks and gives is theoretical justification.

## How does a Permutation Test Work?

We construct an inference method that is valid for small samples sizes. By this we mean that test does not rely on asymptotic behavior of a test statistic. To do so we rely on exchangeability properties arising from the randomization protocol.

The Randomization Protocol is the mechanism that generates treatment status upon selected pre-program variables and a random device that assures stochastic variation of treatment assign ments. We represent this assignment mechanism by a function $\boldsymbol{M}$ whose arguments are measured pre-program variables $X$ and unobserved random variable $R$*.* Specifically,

|  | $D \sim\boldsymbol{M}\left( R,X \right):\mathrm{supp}\left( R \right)\times\mathrm{supp}\left( \boldsymbol{X} \right)\to\mathrm{supp}\left( D \right)$ | (1) |
| --- | --- | --- |

where $0$ “supp” means support. In our notation, variables $X, R$ and $D$ are $N$ -dimensional vectors

whose elements are associated with *N* participants in the sample. Notationally, we write $D = \left( D_{i}; i \in I \right)$ where $i \in I$ and $I = \left\{ 1, . . . , N \right\}$ denotes the sample size. Likewise,$Y = \left( Y_{i}; i \in I \right)$*.*

Properties of the random variable $R$ are: (1) $R$ is assumed to be exogenous, i.e., is not caused by

any other variable; and (2) $R$ has a direct effect on only $D$*.* In other words, any further dependence between *R* and outcomes *Y* must be mediated by $D$*.* The baseline variables *X* consist of variables used in the randomization protocol. In the case of Jamaican intervention, families were matched in pairs based on a selection of pre-program variables discussed in the previous section.

By definition of the randomization protocol $\boldsymbol{M}$*,* it follows that $(Y (1), Y (0)) \boldsymbol{\perp} D | (X, R)$ as

$D$ has no variation conditioned on $X$ and $R$*.* Moreover, by properties of $R$*,* we have that $R$ can

only impact $Y$ through $D$. Thus, by fixing $Y$ on values of $D$*,* that is $\left( Y \left( 0 \right), Y \left( 1 \right) \right)$*,* we eliminate the influence of $R$ on $Y$*.* As a consequence, we must have that counterfactual outcomes $\left( Y \left( 1 \right), Y \left( 0 \right) \right)$ are independent of the random variable $R$ conditional on $X, (Y (1), Y (0)) \boldsymbol{\perp} R | X$*.* In our case, $X$ plays the role of a variable that determines the permutation blocks described below.

Our aim is to test the null hypothesis of no treatment effect. This hypothesis is equivalent to the statement that the conditional counterfactual outcome vectors share the same distribution:

**Hypothesis H-1.** $Y\left( 1 \right){}_{=}^{d}Y (0) | X,$

where =*d* denotes equality in distribution.

Permutation-based tests make inferences about hypothesis [**H-1**](#_bookmark5) by exploring the an exchange- ability property of treatment indicators *D* that arise as a consequence of the randomization protocol. An Exchangeability property arises from the fact that, under the protocol $\boldsymbol{M}$*,* participants are share the same values of $X$ are *indistinguishable*. In other words, if the randomization is generated by ([1](#_bookmark4)), then scrambling the order of the participants sharing the same values on $X$ would not have changed the underlying distribution of the vector of treatment assignments $D$*.* Nevertheless, the generated vector of treatment status would be assigned to different participants, as they were scrambled.

Let $\mathcal{g}_{X}$ be the set of all permutations that permute elements only within each stratum of $X$*,*

then the exchangeability property that arises from the randomization protocol can be stated as:

|  | $gD{}_{=}^{d}\boldsymbol{M}\left( R,gX \right)=\boldsymbol{M}\left( R,X \right){}_{=}^{d}D for any g\in\mathcal{g}_{X}$ $\mathrm{where}\mathcal{g}_{X}=\left\{ g;\pi_{g}\mathcal{:I\to I,}is a bijection and \left( \pi_{g}\left( i \right)=j \right)\Rightarrow\left( X_{i}=X_{j} \right)\forall i\mathcal{\in I} \right\}$ | (2) |
| --- | --- | --- |

An important feature of Exchangeability ([2](#_bookmark6)) is that it relies on limited information on the randomization protocol. It does not require a full specification of the distribution $D$ nor requires any information on the assignment mechanism $\boldsymbol{M}$ itself. As a consequence, Exchangeability ([2](#_bookmark6)) remains valid under compromised randomization that is based only on the information contained in $X$*.* Next section explains how the permutation blocks are defined.

## How to define Permutation Blocks?

A necessary condition to perform a permutation test is to condition on the blocks of variables used in the randomization protocol of the Jamaican intervention. The necessary variables that must be used to construct the permutation blocks are: (1) supplementation treatment, (2) indicator for child age on enrolment, and (3) gender.

The use of child age and gender is based on the randomization protocol, which stratified children according to their sex and whether the child is older than 16 months at enrollment. The use of supplementation treatment is due to the fact that we examine the causal effect of the psychological stimulation only. To do so, we pool the data into two groups. The treatment group consists of all the children that experienced the psychological stimulation and may or may not had the supplementation. The new no-treatment group of all the children that did not experienced the psychological stimulation. These may or may not had the supplementation. By using the indicator of supplementation treatment, we aim to control for this arm of the randomization. We assure that we are comparing children experience the stimulation with those who did not conditional on their supplementation status.

We also use mother education as a variable we condition on, even though its use is not strictly necessary. We use the mother education as this variable suffers from sampling variation generated at the onset of the intervention. The procedure we use to define the blocks of permutations is described below:

- - - 1. We first partition participants according to their maternal education.
      2. We further partition the participants whose mother had low education achievement into those who had supplementation or not.
      3. We then partition each of the last two groups according to the indicator whether the child is older than 16 months at enrolment and gender.

This procedure generates a partition of the sample into eight sets. Each of the sets contains participants from both treatment and the no-treatment group. This feature is particularly important because if a set contains participants only from the treatment or no-treatment group, then the set does not add to the inference on the treatment effect of the intervention. In other words, by defining sets that contain participants from both treatment and no-treatment group, we assure that all available data is used in the inference of treatment effects.

The following tables displays the partitioning of the data into blocks of permutation. The fist column provides the identification number of each participant. The second column provides the treatment status regarding the stimulation arm of the intervention. The Third column displays the mother education measure that is an indicator whether mothers had completed secondary exams. The fourth column indicates if the child received the supplement intervention. The fifth column indicates if the participant is male. The sixth column indicates if the child is older than 16 months at the onset of the intervention and the last column indicates the permutation block that each participant belongs to.

**Identification**

**Number**

**Treatment Mother Supplementation Male**

**Child**

**Status**

**Education**

**Intervention**

**Indicator Age (***>* **16 mo.)**

**Permutation**

**Blocks**

| 172 | 0 | 0 | 0 | 0 | 0 | 1 |
| --- | --- | --- | --- | --- | --- | --- |
| 40 | 1 | 0 | 0 | 0 | 0 | 1 |
| 34 | 1 | 0 | 0 | 0 | 0 | 1 |
| 76 | 0 | 0 | 0 | 0 | 0 | 1 |
| 13 | 0 | 0 | 0 | 0 | 0 | 1 |

| 151 | 1 | 0 | 0 | 0 | 1 | 2 |
| --- | --- | --- | --- | --- | --- | --- |
| 112 | 1 | 0 | 0 | 0 | 1 | 2 |
| 106 | 1 | 0 | 0 | 0 | 1 | 2 |
| 145 | 0 | 0 | 0 | 0 | 1 | 2 |
| 39 | 1 | 0 | 0 | 0 | 1 | 2 |
| 74 | 1 | 0 | 0 | 0 | 1 | 2 |
| 162 | 1 | 0 | 0 | 0 | 1 | 2 |
| 113 | 0 | 0 | 0 | 0 | 1 | 2 |
| 150 | 1 | 0 | 0 | 0 | 1 | 2 |
| 59 | 0 | 0 | 0 | 0 | 1 | 2 |
| 90 | 1 | 0 | 0 | 0 | 1 | 2 |
| 157 | 0 | 0 | 0 | 0 | 1 | 2 |
| 12 | 0 | 0 | 0 | 0 | 1 | 2 |

| 33 | 1 | 0 | 1 | 0 | 0 | 3 |
| --- | --- | --- | --- | --- | --- | --- |
| 123 | 1 | 0 | 1 | 0 | 0 | 3 |
| 57 | 1 | 0 | 1 | 0 | 0 | 3 |
| 37 | 0 | 0 | 1 | 0 | 0 | 3 |
| 140 | 1 | 0 | 1 | 0 | 0 | 3 |
| 14 | 0 | 0 | 1 | 0 | 0 | 3 |

**Identification**

**Number**

**Treatment Mother Supplementation Male**

**Child**

**Status**

**Education**

**Intervention**

**Indicator Age (***>* **16 mo.)**

**Permutation**

**Blocks**

| 46 | 1 | 0 | 1 | 0 | 1 | 4 |
| --- | --- | --- | --- | --- | --- | --- |
| 136 | 0 | 0 | 1 | 0 | 1 | 4 |
| 138 | 0 | 0 | 1 | 0 | 1 | 4 |
| 1 | 0 | 0 | 1 | 0 | 1 | 4 |
| 118 | 0 | 0 | 1 | 0 | 1 | 4 |
| 153 | 0 | 0 | 1 | 0 | 1 | 4 |
| 114 | 1 | 0 | 1 | 0 | 1 | 4 |
| 89 | 0 | 0 | 1 | 0 | 1 | 4 |
| 116 | 1 | 0 | 1 | 0 | 1 | 4 |
| 38 | 1 | 0 | 1 | 0 | 1 | 4 |
| 159 | 1 | 0 | 1 | 0 | 1 | 4 |
| 75 | 0 | 0 | 1 | 0 | 1 | 4 |
| 160 | 1 | 0 | 1 | 0 | 1 | 4 |
| 70 | 1 | 0 | 1 | 0 | 1 | 4 |
| 5 | 0 | 0 | 1 | 0 | 1 | 4 |

| 139 | 1 | 0 | 0 | 1 | 0 | 5 |
| --- | --- | --- | --- | --- | --- | --- |
| 98 | 1 | 0 | 0 | 1 | 0 | 5 |
| 92 | 0 | 0 | 0 | 1 | 0 | 5 |
| 83 | 0 | 0 | 0 | 1 | 0 | 5 |
| 86 | 1 | 0 | 0 | 1 | 0 | 5 |
| 10 | 1 | 0 | 0 | 1 | 0 | 5 |
| 181 | 0 | 0 | 0 | 1 | 0 | 5 |
| 104 | 0 | 0 | 0 | 1 | 0 | 5 |
| 177 | 1 | 0 | 0 | 1 | 1 | 5 |
| 77 | 0 | 0 | 0 | 1 | 1 | 5 |
| 154 | 0 | 0 | 0 | 1 | 1 | 5 |
| 29 | 0 | 0 | 0 | 1 | 1 | 5 |
| 134 | 1 | 0 | 0 | 1 | 1 | 5 |
| 133 | 0 | 0 | 0 | 1 | 1 | 5 |
| 45 | 1 | 0 | 0 | 1 | 1 | 5 |
| 22 | 1 | 0 | 0 | 1 | 1 | 5 |
| 73 | 1 | 0 | 0 | 1 | 1 | 5 |
| 3 | 0 | 0 | 0 | 1 | 1 | 5 |

| 36 | 1 | 0 | 1 | 1 | 0 | 6 |
| --- | --- | --- | --- | --- | --- | --- |
| 25 | 0 | 0 | 1 | 1 | 0 | 6 |
| 109 | 0 | 0 | 1 | 1 | 0 | 6 |
| 178 | 1 | 0 | 1 | 1 | 0 | 6 |
| 47 | 0 | 0 | 1 | 1 | 0 | 6 |
| 84 | 1 | 0 | 1 | 1 | 0 | 6 |

| 142 | 1 | 0 | 1 | 1 | 1 | 7 |
| --- | --- | --- | --- | --- | --- | --- |
| 161 | 1 | 0 | 1 | 1 | 1 | 7 |
| 87 | 0 | 0 | 1 | 1 | 1 | 7 |
| 99 | 1 | 0 | 1 | 1 | 1 | 7 |
| 88 | 0 | 0 | 1 | 1 | 1 | 7 |
| 69 | 1 | 0 | 1 | 1 | 1 | 7 |
| 15 | 1 | 0 | 1 | 1 | 1 | 7 |
| 91 | 1 | 0 | 1 | 1 | 1 | 7 |
| 152 | 0 | 0 | 1 | 1 | 1 | 7 |
| 124 | 1 | 0 | 1 | 1 | 1 | 7 |
| 30 | 1 | 0 | 1 | 1 | 1 | 7 |
| 60 | 1 | 0 | 1 | 1 | 1 | 7 |
| 149 | 0 | 0 | 1 | 1 | 1 | 7 |
| 43 | 0 | 0 | 1 | 1 | 1 | 7 |
| 42 | 1 | 0 | 1 | 1 | 1 | 7 |
| 103 | 1 | 0 | 1 | 1 | 1 | 7 |
| 8 | 0 | 0 | 1 | 1 | 1 | 7 |

**Identification**

**Number**

**Treatment Mother Supplementation Male**

**Child**

**Status**

**Education**

**Intervention**

**Indicator Age (***>* **16 mo.)**

**Permutation**

**Blocks**

| 11 | 1 | 1 | 0 | 0 | 0 | 8 |
| --- | --- | --- | --- | --- | --- | --- |
| 129 | 0 | 1 | 0 | 0 | 1 | 8 |
| 18 | 0 | 1 | 0 | 0 | 1 | 8 |
| 44 | 1 | 1 | 1 | 0 | 0 | 8 |
| 100 | 0 | 1 | 1 | 0 | 1 | 8 |
| 49 | 0 | 1 | 0 | 1 | 0 | 8 |
| 111 | 0 | 1 | 0 | 1 | 1 | 8 |
| 27 | 0 | 1 | 0 | 1 | 1 | 8 |
| 167 | 1 | 1 | 0 | 1 | 1 | 8 |
| 53 | 0 | 1 | 0 | 1 | 1 | 8 |
| 94 | 1 | 1 | 0 | 1 | 1 | 8 |
| 2 | 0 | 1 | 1 | 1 | 0 | 8 |
| 135 | 0 | 1 | 1 | 1 | 1 | 8 |
| 101 | 0 | 1 | 1 | 1 | 1 | 8 |
| 163 | 0 | 1 | 1 | 1 | 1 | 8 |

Increasing the number of blocks of permutations reduces the number of participants that share the same values of the conditioning variables. This may render some permutation blocks invalid as some blocks may contain participants from only the treatment or the no-treatment group. Effectively, we lose those observations as the treatment status does not vary within this block. To avoid this problem, we apply a parsimonious selection of conditioning covariates. Thus, we made sure to check that each cell of analysis has participants from both the treatment and the no-treatment groups.

The treatment effects are conditional on the permutation blocks. The blocks account for the sampling variation of baseline variables described in the previous section. Table S1 presents the difference of baseline variables between treatment and the no-treatment groups conditioned on permutation blocks. As expected, conditioning on these blocks eliminates the few baseline discrepancies between treatment arms.

## The Comparison Sample

The comparison sample consists of children without stunted growth. We found and interviewed 64 children out of the 84 children originally surveyed with an implied attrition rate of 24%, which is slightly lower than that for the experimental sample. The interviewed sample of comparison participants is almost identical to the one examined at age-22 survey. Specifically, at age 22, 65 out of the 84 participants were interviewed. At age 31, only one additional participant was not interviewed.

The properties of the comparison group are described in [Gertler et al.](#_bookmark22) ([2014](#_bookmark22)). There are significant differences in the baseline characteristics of the attrition and non-attrition groups for about a third of baseline variables. Mothers in the attrition group are older, perform better on the Picture Peabody Verbal Test (PPVT), provide more verbal stimulation to their children and live in better houses than mothers of the participants that were interviewed.

# Appendix S3. Description of Empirical Evaluations

This paper estimates the causal effects of the stimulation intervention on psychological outcomes at age 31. We estimate the treatment effects in the experimental stunted sample by linear regression controlling for the variables used in the randomization protocol (supplementation intervention, child age and sex) in addition to mother education. We control for these variables in a non-parametric fashion using a partition of the sample data described in Appendix S2.

*Motivation for Adopting Permutation-based Inference*

Classical inference employs asymptotic theory to describe the distribution of test statistics. The approach is suited for large sample sizes that justify the use of asymptotic distributions. The requirement for the use asymptotic theory does not hold in the Jamaica Study which has a small sample size. We address this problem by using non-parametric permutation tests.

The simplest inference that relies on permutation analysis is the Fisher’s exact test. The test is useful to do inference on the difference between treatment and no-treatment groups for

binary outcomes. The data can be summarised by a 2 *×* 2 contingency table. The inference

generates exact p-values that do not rely on asymptotic arguments. Instead, the test uses the

argument that conditional on the total sum of the outcome, the distribution of its values does not depend on the treatment assignment. The distribution of the possible contingency tables follows the hypergeometric distribution.

Fisher’s exact test can be generalised to account for multiple contingency tables. This is fortunately because we can condition on the permutation cells described in the previous section. Indeed, the structure of the randomization protocol requires us to permute within the strata blocks used for the original randomization. We expand the number of these strata blocks to include the other variables not balanced at baseline.

The Barnard’s exact test and the Cochran–Mantel–Haenszel test are variations of the Fisher’s test that do inference on contingency tables. The Barnard’s exact test allows for random margins while Fisher’s is conditional on the observed data. The Cochran–Mantel–Haenszel, on the other hand, is suitable to do inference on categorical data instead of binary outcomes.

One disadvantage of these tests is that they do not apply to continuous outcomes, which cannot be summarised into contingency tables without loss of information. The counterpart of the Fisher’s exact test for continuous data is the Chi-squared, developed originally by the statistician Karl Pearson. The test is often called a goodness of fit test. The benefit of the test that it is suitable for comparing the distribution of treatment and no-treatment outcomes that are continuous. The disadvantaged of the test is that its *p*-value employs the Chi-squared distribution which relies on the asymptotic behavior of the test statistic.

The Chi-squared test allows for potential stratification across treatment and no-treatment groups. A generalization of the Chi-squared test that compares multiple treatment arms is called Analysis of Variance (ANOVA). The goal of these tests is to test the difference between treatment arms conditional on the data stratification.

In economics, the ANOVA estimation is usually described as a linear regression that uses fixed effects for each of the stratification sets. Formally, let $i\in\left\{ 1, \ldots, N \right\}$ be the index of a participant $i$ of the Jamaican intervention. Let *Y_i_* denote the outcome of participant $i$ and $D_{i}$ denote a treatment

indicator, $D_{i}=1$ if agent $i$ is assigned to the treatment group and $D_{i}=0$ if agent $i$ is assigned to the no-treatment group. We control for the permutation blocks described in Appendix S2. The ANOVA estimation accounts for potential variation of the treatment within each of the strata groups.

We seek to test if the average treatment effect across the strata is positive. In other words, our goal is to conduct a single-sided test whether the treatment effect condition on the strata is positive. Conditioning on the block permutations controls for the imbalance of baseline variables. It enables to evaluate a treatment effect that accounts for the potential bias generated by sampling variation and differential attrition between treatment and no-treatment groups.

We also estimate the outcome mean of the treatment and no-treatment groups conditional on permutation blocks. A suitable statistic to test the presence of treatment effect is the *t*-statistic associated with the he null hypothesis that $\beta$ is equal o zero against the alternative hypothesis that $\beta$ is positive, that is, $H_{0} : \beta= 0$ versus $H_{A} :\beta>0$*.* Another valid statistic is the effect size computed as the Hedges g statistic. We refer to [Rosnow and Rosenthal](#_bookmark29) ([2003](#_bookmark29)) for a discussion on effect sizes.

A disadvantage of using classical test statistics is that they rely of its asymptotic distribution that are unlikely to hold for small sample sizes. Instead, we follow the non-parametric permutation tests described in [Campbell et al.](#_bookmark21) ([2013](#_bookmark21)); [Gertler et al.](#_bookmark22) ([2014](#_bookmark22)); [Heckman et al.](#_bookmark25) ([2010](#_bookmark25)). The test computes the distribution a statistic (either the *t*-statistic or the effect size) generated by multiple permutation draws of the treatment indicator within each of the permutation blocks. The test is valid in small samples because as it does not rely on assumptions about the parametric sampling distribution of the test statistic.

*Multiple-hypothesis Testing*

The presence of multiple outcomes leads to the potential danger of arbitrarily selecting “statistically significant” outcomes where high values of test statistics arise by chance. Testing each hypothesis one at a time with a fixed significance level increases the probability of a type-I error exponentially as the number of outcomes tested grows. We correct for this potential source of bias in inference by performing multiple hypothesis testing based on the Family-Wise Error Rate (FWER), which is the probability of rejecting at least one true null Hypothesis. We use the step- down algorithm proposed in [Romano and Wolf](#_bookmark28) ([2005](#_bookmark28)), which generates inference exhibiting strong FWER control. Associated with each outcome is a single null hypothesis of no treatment effect. We implement the stepdown procedure for blocks of outcomes that share similar meanings.

*Summary Statistic based on the Average Rank Across Outcomes*

In addition to the stepdown procedure, we conduct multiple hypothesis inference based on a non-parametric summary statistic that aggregates multiple outcomes measures. We first transform the outcomes into the rank of each participant for each outcome. We then compute the mean of the rank of each participant across outcomes. We then use the difference in means of participant rank average as a test statistic.

Formally, let $\mathcal{I}$ be the set indexing participants of the Jamaican intervention. Let $D=\left( D_{i}; i\in\mathcal{I} \right)$ be the vector of treatment assignments, such that $D_{i}$ takes value 1 if participant $i$ is assigned to treatment and $0$ otherwise. Let $K = \left\{ 1, . . . , K \right\}$ be an index set for a selection of outcomes sought to be tested, such that $Y_{k} = \left( Y_{i},k; i \in\mathcal{I} \right)$ denotes the vector of $k$-th outcome associated with index $k\in K$*.* Let $Y_{k}$ be the dimension of outcome vector $Y_{k}$*.* In this notation, we can compute the rank of the participants within outcome $k$ by:

|  | $\forall i\mathcal{\in I,}R_{i,k}=\frac{\sum_{j\mathcal{\in I}} 1\left[ Y_{i,k}\geq Y_{j,k} \right]}{\left\vert Y_{k} \right\vert}$ |  |
| --- | --- | --- |

Let the average rank of participant $i\in\mathcal{I}$ across outcomes in $\mathcal{K}$ be:

|  | $\forall i\mathcal{\in I,}R_{i}=\frac{\sum_{k\in\mathcal{K}} R_{i,k}}{\mathcal{\vert K\vert}}$ |  |
| --- | --- | --- |

The vector of the rank average across outcomes in $\mathcal{K}$ for all participants in $\mathcal{I}$*,* that is, $R = (R_{i}; i \in\mathcal{I)}$*,* can be used as a combined measure across outcomes. The associated test statistic comparing

treatment and no-treatment groups is the standard difference in means across treatment groups, namely:

$$\Delta R=\frac{\Sigma_{i\in I}D_{i}R_{i}}{\Sigma_{i\in I}D_{i}}-\frac{\Sigma_{i\in I}\left( {1-D}_{i} \right)R_{i}}{\Sigma_{i\in I}{(1-D}_{i})}.$$

We use permutation methods to obtain the sampling distribution.

The average rank statistics is not included in the stepdown procedure. This means that the hypothesis that the difference-in-means of the rank-mean statistic is equal to zero does not join the inference that jointly tests whether the difference-in-means of the outcomes is equal to zero. There are two reasons for not including the mean rank statistics in the procedure.

The first reason is that stepdown requires a subset pivotality condition. In is useful to consider the case of testing $K + 1$ hypotheses $H_{j}; j \in\{1, ..., K + 1\}$*.* The subset pivotality condition requires that the distribution of the test statistics used for testing the last hypotheses $H_{K+1}$ is unaffected

by the truth or falsehood of the previous hypotheses $H_{j}; j \in\{1, ..., K\}$*.* Including the rank-mean statistics as a new hypothesis in the outcome joint test would violate this condition. Specifically,

consider the inference that jointly test the treatment effects for *K* outcomes. Suppose that that outcomes are measured as ranks. The statistics used to test the treatment effect of the outcomes is the difference-in-means between treatment and no-treatment groups. Let the $(K + 1)$-th hypothesis be the one that uses the rank-mean statistic. If the hypothesis that the treatment group outperforms the no-treatment group in all outcomes 1 through $K$ is true, then it will also affect the inference on the rank-mean. Namely, the outcome rank-mean of the treatment group outcomes should be higher that its counterpart in the no-treatment group. Thereby subset pivotality is violated.

The second reason for not including the rank-mean statistic into the stepdown procedure is that the test that uses the rank-mean statistics is itself a valid joint test. The key difference between the stepdown procedure and the rank-mean test is that the rank-mean employs a summary statistics while the stepdown uses an algorithm. The rank-mean test does not control for FWER while the stepdown does.

*Non-migrants and Gender Comparison*

We examine the concern that the results might be affected by migrants. We also examine the treatment effects of the stimulation arm of the Jamaican intervention separately by gender. We also estimated the treatment effects separately by gender in order to assess gender differences on treatment effects.

Table S2 and S3 re-estimate the effect of treatment on psychological out- comes excluding the migrants and by gender. Table [S2](#_bookmark11) shows the inference on causal effects of the outcomes from Table 2 of the main paper for the subsamples of non-migrants, males and females. Similarly, Table S3 shows the outcomes from Table 3 of the main paper for these sub-samples.

These results should be considered with caution as the study was neither designed nor powered to assess impact separately by gender.

Analyses by gender showed treatment effects on risk taking were in women only (Table S3). There were no other significant gender differences although effect sizes for IQ, social inhibition and

rank mean score for substance use were larger (difference of *≥* 0.5 SD) in males (Tables S2 and

S3).

Regarding migration, we observe that results are qualitatively similar for all data and for the data set that consists of non-migrants only. Regarding gender, we observe that treatment effects on cognition, executive function, mental health, conscientiousness and substance abuse are stronger for males than females. Risk taking behavior is the only outcome where females treatment effects are significantly higher than males.

*Adjustments for Baseline Imbalance*

We assess the extent to which baseline imbalances and attrition affected the treatment effect estimates. As mentioned, correct for potential imbalances of the baseline variables using the method of augmented inverse probability weighting (AIPW). The AIPW is derived from the IPW method that reweighs data using baseline variables to comply with the original distribution of data under no attrition. Both methods, the standard IPW approach and the Augmented IPW, are described in greater detail in the next section.

The basic ideas behind the IPW and the AIPW methods were developed by statisticians almost three decades ago ([Robins](#_bookmark26), [1999](#_bookmark26); [Robins et al.](#_bookmark27), [1994](#_bookmark27); [Scharfstein et al.](#_bookmark30), [1999](#_bookmark30)). The AIPW estimator is less known than the IPW (see [Glynn and Quinn](#_bookmark24) ([2010b](#_bookmark24)) for a recent review on AIPW). Both rely on the evaluation of the propensity score, or the probability of treatment assignment conditional on baseline variables. The AIPW method improves upon the standard IPW estimator as it fully utilizes the information in the baseline variables. Namely, it employs the baseline variables to estimate the propensity score but also use these variables to estimate the outcome itself. In summary, the AIPW estimator exploits the predictive information of conditioning variables at baseline to about the outcome variable while the IPW estimator does not.

We estimate AIPW probabilities using a logit model of treatment assignment and attrition indicator as a function of the baseline characteristics. We use mother education, gender, supplementation arm and child age at enrollment for the AIPW covariates. Tables S4-S5 estimate the same treatment effects of Tables 2–3 of the main paper using the AIPW method. Our results show that the attrition correction does not imply any qualitative change in our main results.

In the same fashion, Tables S6-S7 estimate the same treatment effects as Tables S2–S3 r using the AIPW method. Results remain qualitatively similar to the results presented in the paper regardless of the correction. Thereby we conclude that attrition is not of main concern.

*Examining Treatment Groups with the Comparison Group*

We examine the hypothesis whether the treated could make treatment participants catchup with the participants in the comparison group. To do so, we compare the group of children without stunted growth (the comparison group) with the no-treatment and treatment groups separately.

Tables S8 and S10 investigate the same set of outcomes displayed in Table 2 of the main paper. Table [S8](#_bookmark17) compares the no-treatment participants with the comparison group that did not present stunted growth. Table [S10](#_bookmark19) compares the treatment group with the comparison group . t.

Tables S9 andS11 investigate the same set of outcomes displayed in Table 3 of the main paper. Table S9 compares the no-treatment participants with the comparison group that consists of children that did not present stunted growth. Table S11 compares the treatment group with the comparison group .

14

### Table S1. Conditional Block Permutation Inference on Participants Characteristics Using t-statistic - All Data

| **Reverse** | **Sample** | **No-treatment** | **Treatment** | **Treat.** | **Effect** | **Asymptotic** (two-sided) |
| --- | --- | --- | --- | --- | --- | --- |
| **Variable** | **#C #T** | **Mean Std.Dev.** | **Mean Std.Dev.** | **Effects** | **Size** | *t***-stat Single** *p***-val** |

*Child characteristics on Enrolment*

| Enrollment Age in Months | No | 47 | 48 | 19*.*21 | 3*.*38 | 18*.*94 | 2*.*88 | *−*0*.*27 | *−*0*.*08 | *−*0*.*39 | 0*.*694 |
| --- | --- | --- | --- | --- | --- | --- | --- | --- | --- | --- | --- |
| Gender | No | 47 | 48 | 1*.*45 | 0*.*22 | 1*.*48 | 0*.*15 | 0*.*03 | 0*.*14 | 0*.*72 | 0*.*473 |
| Birth Weight | No | 47 | 47 | 2*.*96 | 0*.*44 | 2*.*81 | 0*.*48 | *−*0*.*15 | *−*0*.*34 | *−*1*.*45 | 0*.*152 |
| Z-score (Weight for Height) at Onset | No | 47 | 48 | *−*0*.*91 | 0*.*70 | *−*1*.*16 | 0*.*66 | *−*0*.*25 | *−*0*.*35 | *−*1*.*64 | 0*.*104 |
| Height-for-age | No | 47 | 48 | *−*2*.*89 | 0*.*64 | *−*2*.*96 | 0*.*48 | *−*0*.*07 | *−*0*.*11 | *−*0*.*59 | 0*.*557 |
| Enrollment DQ | No | 47 | 48 | 96*.*85 | 7*.*94 | 99*.*23 | 7*.*80 | 2*.*38 | 0*.*30 | 1*.*37 | 0*.*174 |
| Housing Score at Onset | No | 47 | 48 | 7*.*43 | 1*.*73 | 7*.*31 | 1*.*39 | *−*0*.*12 | *−*0*.*07 | *−*0*.*35 | 0*.*724 |
| *Parental Characteristics on Enrolment* |  |  |  |  |  |  |  |  |  |  |  |
| HOME Score at Onset | No | 47 | 48 | 16*.*75 | 4*.*63 | 16*.*12 | 3*.*63 | *−*0*.*63 | *−*0*.*14 | *−*0*.*69 | 0*.*494 |
| Mothers PPVT at Onset | No | 47 | 48 | 83*.*75 | 15*.*03 | 87*.*10 | 20*.*98 | 3*.*35 | 0*.*22 | 0*.*83 | 0*.*409 |
| Young Mother Indicator | No | 47 | 48 | 0*.*25 | 0*.*41 | 0*.*24 | 0*.*39 | *−*0*.*01 | *−*0*.*02 | *−*0*.*09 | 0*.*929 |
| Mother Education Indicator | No | 47 | 48 | 0*.*16 | 0*.*00 | 0*.*16 | 0*.*00 | 0*.*00 | 0*.*00 | – | – |
| Mother Working Indicator | No | 47 | 48 | 0*.*19 | 0*.*36 | 0*.*23 | 0*.*42 | 0*.*05 | 0*.*14 | 0*.*57 | 0*.*570 |
| *Follow-up Characteristics* |  |  |  |  |  |  |  |  |  |  |  |
| age at 30year | No | 47 | 48 | 31*.*78 | 0*.*35 | 31*.*80 | 0*.*33 | 0*.*02 | 0*.*06 | 0*.*28 | 0*.*778 |
| Migrant Indicator at age 30 | No | 47 | 48 | 0*.*16 | 0*.*33 | 0*.*18 | 0*.*36 | 0*.*02 | 0*.*06 | 0*.*27 | 0*.*790 |
| factor score water toilet crowding possessions | No | 47 | 47 | *−*0*.*16 | 0*.*94 | 0*.*12 | 0*.*86 | 0*.*27 | 0*.*29 | 1*.*38 | 0*.*171 |

The columns of this table presents the following information. **Col.1**: variable of interest; **Col.2**: indicates if the variable is reverse, that is multiplied by -1 in order to report desired positive treatment effects ; **Col.3**: No-treatment group sample; **Col.4**: Treatment group sample; **Col.5**: No-treatment mean; **Col.6**: standard deviation for the no-treatment group; **Col.7**: treatment mean; **Col.8**: standard deviation for the treatment group; **Col.9**: estimated treatment effect; **Col.10**: Hedges g effect size according to Rosenthal and Rosnow (1991) and Becker (2000). **Col.11**: t-statistic associated with the treatment effect. **Col.12**: asymptotic one-sided *p*-value for the single hypothesis testing of no difference between the treatment and no-treatment means. Estimates in this table are conditioned on main baseline variables used in the randomization protocol.

### Table S2. Treatment Effects on Cognitive, Psychosocial and Personality Outcomes for Non-migrants and by Gender

15

**Non-migrants (Sample** *NC* =**39***, NT* =**40)**

**Males (Sample** *NC* =**26***, NT* =**25)**

**Females (Sample** *NC* =**21***, NT* =**23) Gender Difference**

**Reverse Diff. Effect Permut.** *p***-values Diff. Effect Permut.** *p***-values Diff. Effect Permut.** *p***-values** **Diff. Perm.**

**Variable Means Size Single Joint Means Size Single Joint Means Size Single Joint Means** *p***-val.**

*WASI IQ Scores*

| Full Scale IQ Score | No | 8*.*43 | 0*.*80 | **0***.***00** | **0***.***00** | 9*.*33 | 0*.*78 | **0***.***01** | **0***.***01** | 2*.*49 | 0*.*21 | 0*.*23 | 0*.*28 | 6*.*84 | 0*.*17 |
| --- | --- | --- | --- | --- | --- | --- | --- | --- | --- | --- | --- | --- | --- | --- | --- |
| Perceptual Reasoning | No | 8*.*41 | 0*.*74 | **0***.***00** | **0***.***01** | 8*.*91 | 0*.*70 | **0***.***01** | **0***.***02** | 3*.*37 | 0*.*26 | 0*.*18 | 0*.*26 | 5*.*55 | 0*.*29 |
| Verbal Composite Score | No | 7*.*57 | 0*.*63 | **0***.***01** | **0***.***01** | 8*.*18 | 0*.*58 | **0***.***02** | **0***.***02** | 1*.*64 | 0*.*12 | 0*.*32 | 0*.*32 | 6*.*54 | 0*.*23 |
| Rank Mean | No | 0*.*17 | 0*.*73 | **0***.***01** | – | 0*.*16 | 0*.*66 | **0***.***02** | – | 0*.*06 | 0*.*24 | 0*.*23 | – | 0*.*10 | 0*.*37 |
| *Executive Function* |  |  |  |  |  |  |  |  |  |  |  |  |  |  |  |
| Fluency: Switching Accuracy | No | 0*.*87 | 0*.*31 | 0*.*15 | 0*.*15 | 1*.*12 | 0*.*40 | 0*.*14 | 0*.*24 | *−*0*.*01 | *−*0*.*00 | 0*.*51 | 0*.*51 | 1*.*13 | 0*.*39 |
| Fluency: % Switching Accuracy | No | 1*.*15 | 0*.*35 | **0***.***09** | 0*.*18 | 1*.*55 | 0*.*44 | **0***.***07** | 0*.*16 | 0*.*40 | 0*.*11 | 0*.*34 | 0*.*45 | 1*.*15 | 0*.*40 |
| Card Sorting Total Score | No | 1*.*98 | 0*.*74 | **0***.***00** | **0***.***02** | 1*.*90 | 0*.*63 | **0***.***02** | **0***.***07** | 1*.*39 | 0*.*46 | **0***.***04** | 0*.*14 | 0*.*50 | 0*.*68 |
| Card Sort Description | No | 1*.*80 | 0*.*70 | **0***.***01** | **0***.***03** | 1*.*76 | 0*.*62 | **0***.***03** | **0***.***09** | 1*.*16 | 0*.*41 | **0***.***07** | 0*.*20 | 0*.*60 | 0*.*62 |
| Tower Achievement Score | No | 0*.*84 | 0*.*25 | 0*.*13 | 0*.*21 | 0*.*59 | 0*.*16 | 0*.*25 | 0*.*25 | 1*.*00 | 0*.*27 | 0*.*11 | 0*.*24 | *−*0*.*41 | 0*.*73 |
| Rank Mean | No | 0*.*12 | 0*.*57 | **0***.***01** | – | 0*.*13 | 0*.*62 | **0***.***02** | – | 0*.*07 | 0*.*33 | 0*.*13 | – | 0*.*06 | 0*.*50 |
| *Mental Health* |  |  |  |  |  |  |  |  |  |  |  |  |  |  |  |
| Depressive symptoms | Yes | 5*.*65 | 0*.*59 | **0***.***01** | **0***.***03** | 6*.*08 | 0*.*76 | **0***.***01** | **0***.***03** | 5*.*16 | 0*.*65 | **0***.***06** | 0*.*14 | 0*.*92 | 0*.*82 |
| Anxiety Total Score | Yes | 2*.*09 | 0*.*26 | 0*.*13 | 0*.*13 | 2*.*52 | 0*.*32 | 0*.*11 | 0*.*11 | 3*.*27 | 0*.*42 | 0*.*11 | 0*.*20 | *−*0*.*75 | 0*.*83 |
| Social inhibition | Yes | 1*.*46 | 0*.*44 | **0***.***04** | **0***.***07** | 2*.*28 | 0*.*77 | **0***.***01** | **0***.***02** | 0*.*12 | 0*.*04 | 0*.*46 | 0*.*46 | 2*.*17 | 0*.*15 |
| Rank Mean | No | 0*.*12 | 0*.*55 | **0***.***01** | – | 0*.*18 | 0*.*88 | **0***.***00** | – | 0*.*08 | 0*.*39 | 0*.*15 | – | 0*.*10 | 0*.*30 |
| *Psychosocial Skills* |  |  |  |  |  |  |  |  |  |  |  |  |  |  |  |
| Self-esteem | No | 1*.*49 | 0*.*28 | 0*.*12 | 0*.*21 | *−*0*.*18 | *−*0*.*03 | 0*.*55 | 0*.*55 | 2*.*39 | 0*.*46 | **0***.***07** | 0*.*13 | *−*2*.*56 | 0*.*23 |
| Grit | No | 2*.*50 | 0*.*54 | **0***.***01** | **0***.***04** | 1*.*46 | 0*.*36 | 0*.*11 | 0*.*18 | 3*.*06 | 0*.*75 | **0***.***01** | **0***.***04** | *−*1*.*60 | 0*.*38 |
| Self-control | No | 0*.*66 | 0*.*21 | 0*.*21 | 0*.*21 | 1*.*36 | 0*.*43 | **0***.***07** | 0*.*19 | 0*.*05 | 0*.*02 | 0*.*48 | 0*.*48 | 1*.*31 | 0*.*35 |
| Rank Mean  *Personality Traits* | No | 0*.*10 | 0*.*45 | **0***.***03** | – | 0*.*07 | 0*.*32 | 0*.*12 | – | 0*.*11 | 0*.*49 | **0***.***06** | – | *−*0*.*04 | 0*.*68 |
| Extraversion | No | 0*.*29 | 0*.*11 | 0*.*33 | 0*.*52 | 0*.*11 | 0*.*06 | 0*.*43 | 0*.*43 | 0*.*24 | 0*.*13 | 0*.*40 | 0*.*74 | *−*0*.*13 | 0*.*91 |
| Agreeableness | No | 0*.*76 | 0*.*32 | 0*.*11 | 0*.*36 | 1*.*06 | 0*.*48 | **0***.***06** | 0*.*20 | *−*0*.*79 | *−*0*.*36 | 0*.*82 | 0*.*82 | 1*.*85 | **0***.***09** |
| Conscientious | No | 2*.*47 | 0*.*87 | **0***.***00** | **0***.***00** | 2*.*17 | 0*.*76 | **0***.***00** | **0***.***01** | 1*.*41 | 0*.*49 | **0***.***03** | 0*.*13 | 0*.*77 | 0*.*46 |
| Emotional Stability | No | 0*.*50 | 0*.*18 | 0*.*23 | 0*.*51 | 0*.*22 | 0*.*09 | 0*.*39 | 0*.*64 | 0*.*47 | 0*.*18 | 0*.*30 | 0*.*68 | *−*0*.*25 | 0*.*83 |
| Open to Experiences | No | *−*0*.*17 | *−*0*.*08 | 0*.*64 | 0*.*64 | 0*.*61 | 0*.*26 | 0*.*20 | 0*.*47 | *−*0*.*64 | *−*0*.*27 | 0*.*78 | 0*.*94 | 1*.*25 | 0*.*26 |
| Rank Mean | No | 0*.*09 | 0*.*59 | **0***.***01** | – | 0*.*09 | 0*.*64 | **0***.***02** | – | 0*.*02 | 0*.*16 | 0*.*32 | – | 0*.*07 | 0*.*30 |

The columns of this table presents the following information. **Col.1**: variable of interest; **Col.2**: indicates if the variable is reverse, that is multiplied by -1 in order to report desired positive treatment effects; **Col.3**: estimated treatment effect for nonmigrants; **Col.4**: Hedges g effect size for non-migrants. **Col.5**: the single hypothesis one-sided mid-p-value based on 15.000 permutations draws. Test statistic uses the pre-pivoted treatment effect estimate and the permutation scheme is either a na¨ıve or block permutation. **Col.6**: the multiple joint hypothesis testing (stepdown) for one-sided *p*-values for non-migrants. **Col.7**: treatment effect for males; **Col.8**: effect size for males; **Col.9**: one-sided mid-p-value for males; **Col.10**: stepdown joint hypothesis one-sided *p*-values for males. **Col.11**: treatment effect for females; **Col.12**: effect size for females; **Col.13**: mid-p-value for females; **Col.14**: stepdown joint hypothesis one-sided *p*-values for females. **Col.15**: gender difference of treatment effects; **Col.16**: permutation single hypothesis testing two-sided *p*-values. Estimates are based on a block permutation inference conditional on main variables at the onset of the intervention.

16

### Table S3. Treatment Effects on Risk Taking and Violence Behaviors for Non-migrants and by Gender

|  | **Reverse** | **Non-migrants (Sample** *NC* =**39***, NT* =**40) Diff. Effect Permut.** *p***-values** | | | | **Males (Sample** *NC* =**26***, NT* =**25) Diff. Effect Permut.** *p***-values** | | | | **Females (Sample** *NC* =**21***, NT* =**23) Diff. Effect Permut.** *p***-values** | | | | **Gender** **Difference Diff. Perm.** | |
| --- | --- | --- | --- | --- | --- | --- | --- | --- | --- | --- | --- | --- | --- | --- | --- |
|  | **Variable** | **Means** | **Size** | **Single** | **Joint** | **Means** | **Size** | **Single** | **Joint** | **Means** | **Size** | **Single** | **Joint** | **Means** | *p***-val.** |
| *Substance Abuse (WHO)* |  |  |  |  |  |  |  |  |  |  |  |  |  |  |  |
| Alcohol Usage | Yes | 1*.*12 | 0*.*39 | **0***.***08** | **0***.***08** | 1*.*45 | 0*.*54 | **0***.***09** | **0***.***09** | 0*.*61 | 0*.*23 | 0*.*24 | 0*.*40 | 0*.*84 | 0*.*53 |
| Ganja (Cannabis) Usage | Yes | 1*.*37 | 0*.*37 | **0***.***08** | 0*.*14 | 2*.*13 | 0*.*49 | **0***.***07** | 0*.*12 | 0*.*03 | 0*.*01 | 0*.*44 | 0*.*44 | 2*.*10 | 0*.*19 |
| Rank Mean | No | 0*.*09 | 0*.*48 | **0***.***04** | – | 0*.*14 | 0*.*69 | **0***.***02** | – | 0*.*02 | 0*.*11 | 0*.*35 | – | 0*.*12 | 0*.*19 |
| *Violent Behavior* |  |  |  |  |  |  |  |  |  |  |  |  |  |  |  |
| Fights & Weapons | Yes | 0*.*35 | 0*.*35 | **0***.***04** | **0***.***07** | 0*.*21 | 0*.*22 | 0*.*18 | 0*.*34 | 0*.*47 | 0*.*49 | **0***.***06** | 0*.*12 | *−*0*.*26 | 0*.*49 |
| Guns & Gangs | Yes | *−*0*.*26 | *−*0*.*95 | 1*.*00 | 1*.*00 | *−*0*.*06 | *−*0*.*09 | 0*.*62 | 0*.*62 | *−*0*.*19 | *−*0*.*30 | 0*.*97 | 0*.*97 | 0*.*13 | 0*.*57 |
| Rank Mean  *Risk Taking* | No | *−*0*.*02 | *−*0*.*40 | 0*.*84 | – | *−*0*.*02 | *−*0*.*22 | 0*.*68 | – | *−*0*.*01 | *−*0*.*17 | 0*.*52 | – | *−*0*.*00 | 0*.*84 |
| General Risk & Finance | Yes | 0*.*29 | 0*.*36 | 0*.*11 | 0*.*11 | *−*0*.*17 | *−*0*.*22 | 0*.*74 | 0*.*74 | 0*.*58 | 0*.*75 | **0***.***02** | **0***.***02** | *−*0*.*75 | **0***.***06** |
| Health, Work & Trust | Yes | 0*.*49 | 0*.*51 | **0***.***02** | **0***.***05** | 0*.*15 | 0*.*15 | 0*.*29 | 0*.*45 | 1*.*06 | 1*.*11 | **0***.***00** | **0***.***00** | *−*0*.*91 | **0***.***03** |
| Rank Mean | No | 0*.*10 | 0*.*49 | **0***.***05** | – | *−*0*.*01 | *−*0*.*05 | 0*.*56 | – | 0*.*21 | 1*.*19 | **0***.***00** | – | *−*0*.*22 | **0***.***02** |

The columns of this table presents the following information. **Col.1**: variable of interest; **Col.2**: indicates if the variable is reverse, that is multiplied by -1 in order to report desired positive treatment effects; **Col.3**: estimated treatment effect for nonmigrants; **Col.4**: Hedges g effect size for non-migrants. **Col.5**: the single hypothesis one-sided mid-p-value based on 15.000 permutations draws. Test statistic uses the pre-pivoted treatment effect estimate and the permutation scheme is either a na¨ıve or block permutation. **Col.6**: the multiple joint hypothesis testing (stepdown) for one-sided *p*-values for non-migrants. **Col.7**: treatment effect for males; **Col.8**: effect size for males; **Col.9**: one-sided mid-p-value for males; **Col.10**: stepdown joint hypothesis one-sided *p*-values for males. **Col.11**: treatment effect for females; **Col.12**: effect size for females; **Col.13**: mid-p-value for females; **Col.14**: stepdown joint hypothesis one-sided *p*-values for females. **Col.15**: gender difference of treatment effects; **Col.16**: permutation single hypothesis testing two-sided *p*-values. Estimates are based on a block permutation inference conditional on main variables at the onset of the intervention.

### Table S4. AIPW Inference on Cognitive, Psychosocial and Personality Outcomes Conditional All Data

17

|  | **Reverse** | **No-treatment (n=47)** | **Treatment (n=48)** | **Treated** | **Effect** | **Asymptotic** (one-sided) | **Permutation** (one-sided) |
| --- | --- | --- | --- | --- | --- | --- | --- |
|  | **Variable** | **Mean Std.Dev.** | **Mean Std.Dev.** | **Effects** | **Size** | *t***-stat Single** *p***-val** | **Single** *p***-val Stepdown** |

*WASI IQ Scores*

| Full Scale IQ Score | No | 73*.*02 | 11*.*86 | 77*.*35 | 11*.*44 | 4*.*33 | 0*.*37 | 1*.*83 | **0***.***03** | **0***.***01** | **0***.***01** |
| --- | --- | --- | --- | --- | --- | --- | --- | --- | --- | --- | --- |
| Perceptual Reasoning | No | 72*.*66 | 11*.*75 | 77*.*85 | 12*.*64 | 5*.*19 | 0*.*44 | 2*.*11 | **0***.***02** | **0***.***01** | **0***.***01** |
| Verbal Composite Score | No | 77*.*87 | 14*.*03 | 80*.*75 | 12*.*80 | 2*.*88 | 0*.*21 | 1*.*04 | 0*.*15 | **0***.***03** | **0***.***03** |
| Rank Mean | No | 0*.*46 | 0*.*24 | 0*.*54 | 0*.*27 | 0*.*08 | 0*.*33 | 1*.*52 | **0***.***07** | **0***.***03** | – |
| *Executive Function* |  |  |  |  |  |  |  |  |  |  |  |
| Fluency: Switching Accuracy | No | 8*.*26 | 2*.*93 | 8*.*67 | 3*.*06 | 0*.*41 | 0*.*14 | 0*.*65 | 0*.*26 | **0***.***10** | 0*.*18 |
| Fluency: % Switching Accuracy | No | 8*.*57 | 3*.*38 | 9*.*48 | 3*.*10 | 0*.*90 | 0*.*27 | 1*.*40 | **0***.***08** | **0***.***05** | 0*.*13 |
| Card Sorting Total Score | No | 4*.*55 | 2*.*80 | 5*.*94 | 3*.*08 | 1*.*38 | 0*.*49 | 2*.*29 | **0***.***01** | **0***.***01** | **0***.***04** |
| Card Sort Description | No | 4*.*62 | 2*.*78 | 5*.*81 | 3*.*00 | 1*.*20 | 0*.*43 | 1*.*90 | **0***.***03** | **0***.***01** | **0***.***05** |
| Tower Achievement Score | No | 8*.*53 | 3*.*24 | 9*.*23 | 2*.*00 | 0*.*70 | 0*.*22 | 1*.*25 | 0*.*11 | **0***.***10** | **0***.***10** |
| Rank Mean | No | 0*.*46 | 0*.*20 | 0*.*54 | 0*.*19 | 0*.*08 | 0*.*41 | 2*.*13 | **0***.***02** | **0***.***01** | – |
| *Mental Health* |  |  |  |  |  |  |  |  |  |  |  |
| Depressive symptoms | Yes | *−*20*.*17 | 10*.*46 | *−*15*.*31 | 9*.*77 | 4*.*86 | 0*.*46 | 2*.*37 | **0***.***01** | **0***.***00** | **0***.***01** |
| Anxiety Total Score | Yes | *−*42*.*94 | 8*.*21 | *−*40*.*71 | 6*.*78 | 2*.*23 | 0*.*27 | 1*.*44 | **0***.***08** | **0***.***02** | **0***.***04** |
| Social inhibition | Yes | *−*6*.*47 | 3*.*21 | *−*5*.*52 | 3*.*29 | 0*.*95 | 0*.*30 | 1*.*37 | **0***.***09** | **0***.***05** | **0***.***05** |
| Rank Mean | No | 0*.*45 | 0*.*21 | 0*.*55 | 0*.*21 | 0*.*10 | 0*.*49 | 2*.*39 | **0***.***01** | **0***.***00** | – |
| *Psychosocial Skills* |  |  |  |  |  |  |  |  |  |  |  |
| Self esteem - Rosenberg Score | No | 21*.*68 | 5*.*22 | 22*.*65 | 4*.*52 | 0*.*96 | 0*.*18 | 1*.*00 | 0*.*16 | **0***.***08** | 0*.*15 |
| Grit score | No | 24*.*55 | 4*.*64 | 26*.*56 | 3*.*56 | 2*.*01 | 0*.*43 | 2*.*39 | **0***.***01** | **0***.***00** | **0***.***01** |
| Self-control Measure | No | 9*.*17 | 3*.*37 | 9*.*83 | 2*.*96 | 0*.*66 | 0*.*20 | 0*.*99 | 0*.*16 | **0***.***08** | **0***.***08** |
| Rank Mean | No | 0*.*46 | 0*.*22 | 0*.*54 | 0*.*20 | 0*.*08 | 0*.*38 | 1*.*84 | **0***.***03** | **0***.***02** | – |
| *Personality Traits* |  |  |  |  |  |  |  |  |  |  |  |
| Extraversion | No | 8*.*51 | 2*.*38 | 8*.*44 | 2*.*75 | *−*0*.*07 | *−*0*.*03 | *−*0*.*14 | 0*.*56 | 0*.*34 | 0*.*34 |
| Agreeableness | No | 11*.*77 | 2*.*39 | 11*.*73 | 2*.*54 | *−*0*.*04 | *−*0*.*02 | *−*0*.*07 | 0*.*53 | 0*.*31 | 0*.*65 |
| Conscientious | No | 11*.*17 | 2*.*59 | 12*.*94 | 1*.*76 | 1*.*77 | 0*.*68 | 3*.*96 | **0***.***00** | **0***.***00** | **0***.***00** |
| Emotional Stability | No | 9*.*89 | 2*.*78 | 10*.*27 | 2*.*44 | 0*.*38 | 0*.*14 | 0*.*68 | 0*.*25 | 0*.*21 | 0*.*56 |
| Open to Experiences | No | 10*.*49 | 2*.*31 | 10*.*50 | 2*.*67 | 0*.*01 | 0*.*00 | 0*.*02 | 0*.*49 | 0*.*31 | 0*.*50 |
| Rank Mean | No | 0*.*47 | 0*.*15 | 0*.*53 | 0*.*14 | 0*.*05 | 0*.*35 | 1*.*73 | **0***.***04** | **0***.***05** | – |

The columns of this table presents the following information. **Col.1**: variable of interest; **Col.2**: indicates if the variable is reverse, that is multiplied by -1 in order to report desired positive treatment effects ; **Col.3**: No-treatment group mean; **Col.4**: standard deviation; **Col.5**: Treatment group mean; **Col.6**: standard deviation; **Col.7**: estimated difference in means between participants; **Col.8**: Hedges g effect size according to Rosenthal and Rosnow (1991) and Becker (2000). **Col.9**: t-statistic associated with the treatment effect. **Col.10**: asymptotic one-sided *p*-value for the single hypothesis testing of no difference between treatment groups. **Col.11**: the single hypothesis one-sided mid-p-value based on 15.000 permutations draws. Test statistic uses the pre-pivoted treatment effect estimate and the permutation scheme is either a na¨ıve or block permutation. **Col.12**: the multiple hypothesis testing (stepdown) for *p*-values in column 10. Estimates are based on a block permutation inference conditional on main variables at the onset of the intervention.

18

### Table S5. AIPW Inference on Risk Taking and Violence Behaviors Conditional All Data

|  | **Reverse** | **No-treatment (n=47)** | **Treatment (n=48)** | **Treated** | **Effect** | **Asymptotic** (one-sided) | **Permutation** (one-sided) |
| --- | --- | --- | --- | --- | --- | --- | --- |
|  | **Variable** | **Mean Std.Dev.** | **Mean Std.Dev.** | **Effects** | **Size** | *t***-stat Single** *p***-val** | **Single** *p***-val Stepdown** |

*Substance Abuse (WHO)*

| Aggregate Alcohol Score | Yes | *−*3*.*43 | 3*.*05 | *−*2*.*67 | 3*.*20 | 0*.*76 | 0*.*25 | 1*.*20 | 0*.*12 | **0***.***07** | 0*.*13 |
| --- | --- | --- | --- | --- | --- | --- | --- | --- | --- | --- | --- |
| Aggregate Ganja Score (WHO) | Yes | *−*2*.*94 | 4*.*63 | *−*2*.*31 | 3*.*70 | 0*.*62 | 0*.*13 | 0*.*70 | 0*.*24 | **0***.***08** | **0***.***08** |
| Rank Mean | No | 0*.*47 | 0*.*24 | 0*.*53 | 0*.*21 | 0*.*06 | 0*.*25 | 1*.*34 | **0***.***09** | **0***.***02** | – |
| *Risks Taking Factor Scores* |  |  |  |  |  |  |  |  |  |  |  |
| General Risk & Finance | Yes | *−*3*.*89 | 0*.*90 | *−*3*.*70 | 1*.*15 | 0*.*20 | 0*.*22 | 0*.*92 | 0*.*18 | 0*.*29 | 0*.*29 |
| Health, Work & Trust | Yes | *−*3*.*23 | 0*.*93 | *−*2*.*68 | 1*.*09 | 0*.*55 | 0*.*59 | 2*.*59 | **0***.***01** | **0***.***00** | **0***.***01** |
| Rank Mean | No | 0*.*45 | 0*.*17 | 0*.*55 | 0*.*25 | 0*.*10 | 0*.*56 | 2*.*11 | **0***.***02** | **0***.***02** | – |
| *Violence Factor Scores* |  |  |  |  |  |  |  |  |  |  |  |
| Factor Score Fights & Weapons | Yes | *−*0*.*05 | 1*.*04 | 0*.*18 | 0*.*50 | 0*.*23 | 0*.*22 | 1*.*37 | **0***.***09** | **0***.***05** | 0*.*11 |
| Factor Score Guns & Gangs | Yes | 0*.*30 | 0*.*48 | 0*.*18 | 0*.*58 | *−*0*.*12 | *−*0*.*25 | *−*1*.*17 | 0*.*88 | 0*.*86 | 0*.*86 |
| Rank Mean | No | 0*.*51 | 0*.*05 | 0*.*49 | 0*.*13 | *−*0*.*02 | *−*0*.*46 | *−*1*.*22 | 0*.*89 | 0*.*73 | – |

The columns of this table presents the following information. **Col.1**: variable of interest; **Col.2**: indicates if the variable is reverse, that is multiplied by -1 in order to report desired positive treatment effects ; **Col.3**: No-treatment group mean; **Col.4**: standard deviation; **Col.5**: Treatment group mean; **Col.6**: standard deviation; **Col.7**: estimated difference in means between participants; **Col.8**: Hedges g effect size according to Rosenthal and Rosnow (1991) and Becker (2000). **Col.9**: t-statistic associated with the treatment effect. **Col.10**: asymptotic one-sided *p*-value for the single hypothesis testing of no difference between treatment groups. **Col.11**: the single hypothesis one-sided mid-p-value based on 15.000 permutations draws. Test statistic uses the pre-pivoted treatment effect estimate and the permutation scheme is either a na¨ıve or block permutation. **Col.12**: the multiple hypothesis testing (stepdown) for *p*-values in column 10. Estimates are based on a block permutation inference conditional on main variables at the onset of the intervention.

### Table S6. AIPW Inference on Cognitive, Psychosocial and Personality Outcomes Conditional Summary

19

|  | **Reverse Variable** | **Non-migrants (Sample** *NC* =**39***, NT* =**40) Treat. Effect Permutation**  **Effect Size** *p***-val Stepdown** | **Males (Sample** *NC* =**26***, NT* =**25) Treat. Effect Permutation**  **Effect Size** *p***-val Stepdown** | **Females (Sample** *NC* =**21***, NT* =**23) Treat. Effect Permutation**  **Effect Size** *p***-val Stepdown** |
| --- | --- | --- | --- | --- |

*WASI IQ Scores*

| Full Scale IQ Score | No | 4*.*82 | 0*.*40 | **0***.***00** | **0***.***00** | 6*.*67 | 0*.*53 | **0***.***01** | **0***.***01** | 1*.*59 | 0*.*19 | 0*.*27 | 0*.*32 |
| --- | --- | --- | --- | --- | --- | --- | --- | --- | --- | --- | --- | --- | --- |
| Perceptual Reasoning | No | 5*.*20 | 0*.*44 | **0***.***00** | **0***.***01** | 7*.*95 | 0*.*66 | **0***.***01** | **0***.***03** | 2*.*06 | 0*.*20 | 0*.*19 | 0*.*28 |
| Verbal Composite Score | No | 3*.*88 | 0*.*29 | **0***.***01** | **0***.***01** | 4*.*18 | 0*.*25 | **0***.***02** | **0***.***02** | 1*.*24 | 0*.*14 | 0*.*40 | 0*.*40 |
| Rank Mean | No | 0*.*09 | 0*.*36 | **0***.***01** | – | 0*.*11 | 0*.*42 | **0***.***02** | – | 0*.*04 | 0*.*19 | 0*.*22 | – |
| *Executive Function* |  |  |  |  |  |  |  |  |  |  |  |  |  |
| Fluency: Switching Accuracy | No | 0*.*47 | 0*.*15 | **0***.***09** | 0*.*15 | 0*.*70 | 0*.*25 | **0***.***08** | 0*.*14 | *−*0*.*01 | *−*0*.*00 | 0*.*42 | 0*.*42 |
| Fluency: % Switching Accuracy | No | 0*.*98 | 0*.*28 | **0***.***08** | 0*.*16 | 1*.*30 | 0*.*36 | **0***.***05** | 0*.*15 | 0*.*45 | 0*.*15 | 0*.*25 | 0*.*46 |
| Card Sorting Total Score | No | 1*.*46 | 0*.*52 | **0***.***01** | **0***.***03** | 1*.*52 | 0*.*48 | **0***.***04** | 0*.*14 | 1*.*17 | 0*.*46 | **0***.***07** | 0*.*21 |
| Card Sort Description | No | 1*.*29 | 0*.*46 | **0***.***01** | **0***.***04** | 1*.*37 | 0*.*45 | **0***.***05** | 0*.*14 | 0*.*95 | 0*.*41 | **0***.***10** | 0*.*27 |
| Tower Achievement Score | No | 0*.*84 | 0*.*24 | 0*.*13 | 0*.*13 | 0*.*47 | 0*.*14 | 0*.*25 | 0*.*25 | 0*.*98 | 0*.*36 | 0*.*29 | 0*.*45 |
| Rank Mean | No | 0*.*09 | 0*.*44 | **0***.***01** | – | 0*.*09 | 0*.*47 | **0***.***04** | – | 0*.*07 | 0*.*36 | 0*.*13 | – |
| *Mental Health* |  |  |  |  |  |  |  |  |  |  |  |  |  |
| Depressive symptoms | Yes | 4*.*75 | 0*.*46 | **0***.***00** | **0***.***01** | 5*.*50 | 0*.*70 | **0***.***02** | **0***.***03** | 4*.*27 | 0*.*36 | **0***.***06** | 0*.*14 |
| Anxiety Total Score | Yes | 2*.*06 | 0*.*26 | **0***.***10** | **0***.***10** | 2*.*09 | 0*.*27 | **0***.***07** | **0***.***07** | 2*.*55 | 0*.*29 | **0***.***10** | 0*.*17 |
| Social inhibition | Yes | 1*.*16 | 0*.*36 | **0***.***06** | 0*.*12 | 1*.*65 | 0*.*53 | **0***.***01** | **0***.***02** | 0*.*14 | 0*.*04 | 0*.*58 | 0*.*58 |
| Rank Mean | No | 0*.*10 | 0*.*45 | **0***.***01** | – | 0*.*15 | 0*.*73 | **0***.***00** | – | 0*.*06 | 0*.*27 | 0*.*13 | – |
| *Psychosocial Skills* |  |  |  |  |  |  |  |  |  |  |  |  |  |
| Self esteem - Rosenberg Score | No | 1*.*25 | 0*.*22 | **0***.***06** | 0*.*12 | *−*0*.*05 | *−*0*.*01 | 0*.*49 | 0*.*49 | 2*.*08 | 0*.*41 | **0***.***04** | **0***.***07** |
| Grit score | No | 2*.*07 | 0*.*44 | **0***.***01** | **0***.***02** | 1*.*54 | 0*.*38 | **0***.***07** | 0*.*13 | 2*.*50 | 0*.*50 | **0***.***03** | **0***.***09** |
| Self-control Measure | No | 0*.*57 | 0*.*19 | 0*.*23 | 0*.*23 | 1*.*39 | 0*.*45 | **0***.***05** | 0*.*13 | *−*0*.*20 | *−*0*.*06 | 0*.*37 | 0*.*37 |
| Rank Mean | No | 0*.*09 | 0*.*42 | **0***.***04** | – | 0*.*08 | 0*.*33 | 0*.*17 | – | 0*.*08 | 0*.*41 | **0***.***07** | – |
| *Personality Traits* |  |  |  |  |  |  |  |  |  |  |  |  |  |
| Extraversion | No | *−*0*.*11 | *−*0*.*04 | 0*.*27 | 0*.*45 | *−*0*.*31 | *−*0*.*17 | 0*.*46 | 0*.*46 | 0*.*13 | 0*.*05 | 0*.*26 | 0*.*63 |
| Agreeableness | No | 0*.*46 | 0*.*19 | 0*.*12 | 0*.*37 | 0*.*75 | 0*.*34 | **0***.***03** | 0*.*13 | *−*0*.*88 | *−*0*.*38 | 0*.*81 | 0*.*81 |
| Conscientious | No | 2*.*10 | 0*.*79 | **0***.***00** | **0***.***00** | 2*.*04 | 0*.*70 | **0***.***00** | **0***.***02** | 1*.*44 | 0*.*57 | **0***.***03** | 0*.*13 |
| Emotional Stability | No | 0*.*81 | 0*.*27 | 0*.*19 | 0*.*45 | 0*.*23 | 0*.*10 | 0*.*26 | 0*.*45 | 0*.*66 | 0*.*24 | 0*.*36 | 0*.*69 |
| Open to Experiences | No | 0*.*01 | 0*.*00 | 0*.*51 | 0*.*51 | 0*.*57 | 0*.*23 | 0*.*16 | 0*.*40 | *−*0*.*62 | *−*0*.*29 | 0*.*74 | 0*.*93 |
| Rank Mean | No | 0*.*08 | 0*.*53 | **0***.***01** | – | 0*.*08 | 0*.*53 | **0***.***03** | – | 0*.*02 | 0*.*14 | 0*.*32 | – |

The columns of this table presents the following information. **Col.1**: variable of interest; **Col.2**: indicates if the variable is reverse, that is multiplied by -1; **Col.3**: estimated treatment effect for nonmigrants; **Col.4**: Hedges g effect size for non-migrants. **Col.5**: the single hypothesis one-sided mid-p-value based on 15.000 permutations draws. Test statistic uses the pre-pivoted treatment effect estimate and the permutation scheme is either a na¨ıve or block permutation. **Col.6**: the multiple hypothesis testing (stepdown) for *p*-values for non-migrants. **Col.7**: treatment effect for males; **Col.8**: effect size for males; **Col.9**: mid-p-value for males; **Col.10**: stepdown *p*-values for males. **Col.11**: treatment effect for females; **Col.12**: effect size for females; **Col.13**: mid-p-value for females; **Col.14**: stepdown *p*-values for females. Estimates are based on a block permutation inference conditional on main variables at the onset of the intervention.

20

### Table S7. AIPW Inference on Risk Taking and Violence Behaviors Conditional Summary

|  | **Reverse Variable** | **Non-migrants (Sample** *NC* =**39***, NT* =**40) Treat. Effect Permutation**  **Effect Size** *p***-val Stepdown** | **Males (Sample** *NC* =**26***, NT* =**25) Treat. Effect Permutation**  **Effect Size** *p***-val Stepdown** | **Females (Sample** *NC* =**21***, NT* =**23) Treat. Effect Permutation**  **Effect Size** *p***-val Stepdown** |
| --- | --- | --- | --- | --- |

*Substance Abuse (WHO)*

| Aggregate Alcohol Score | Yes | 0*.*59 | 0*.*19 | 0*.*10 | 0*.*10 | 0*.*83 | 0*.*29 | **0***.***10** | **0***.***10** | 0*.*55 | 0*.*20 | 0*.*30 | 0*.*47 |
| --- | --- | --- | --- | --- | --- | --- | --- | --- | --- | --- | --- | --- | --- |
| Aggregate Ganja Score (WHO) | Yes | 0*.*25 | 0*.*05 | **0***.***07** | 0*.*13 | 1*.*17 | 0*.*25 | **0***.***05** | **0***.***10** | *−*0*.*25 | *−*0*.*10 | 0*.*67 | 0*.*67 |
| Rank Mean | No | 0*.*03 | 0*.*14 | **0***.***04** | – | 0*.*10 | 0*.*37 | **0***.***03** | – | 0*.*00 | 0*.*02 | 0*.*38 | – |
| *Risks Taking Factor Scores* |  |  |  |  |  |  |  |  |  |  |  |  |  |
| General Risk & Finance | Yes | 0*.*20 | 0*.*25 | 0*.*22 | 0*.*22 | *−*0*.*08 | *−*0*.*08 | 0*.*76 | 0*.*76 | 0*.*49 | 0*.*61 | **0***.***04** | **0***.***04** |
| Health, Work & Trust | Yes | 0*.*40 | 0*.*43 | **0***.***01** | **0***.***02** | 0*.*25 | 0*.*28 | 0*.*31 | 0*.*47 | 0*.*90 | 0*.*97 | **0***.***00** | **0***.***00** |
| Rank Mean | No | 0*.*07 | 0*.*41 | **0***.***06** | – | 0*.*01 | 0*.*07 | 0*.*58 | – | 0*.*18 | 1*.*03 | **0***.***00** | – |
| *Violence Factor Scores* |  |  |  |  |  |  |  |  |  |  |  |  |  |
| Factor Score Fights & Weapons | Yes | 0*.*28 | 0*.*27 | **0***.***08** | 0*.*16 | 0*.*07 | 0*.*08 | 0*.*20 | 0*.*36 | 0*.*42 | 0*.*37 | 0*.*13 | 0*.*25 |
| Factor Score Guns & Gangs | Yes | *−*0*.*24 | *−*0*.*90 | 1*.*00 | 1*.*00 | *−*0*.*09 | *−*0*.*15 | 0*.*62 | 0*.*62 | *−*0*.*17 | *−*0*.*64 | 0*.*96 | 0*.*96 |
| Rank Mean | No | *−*0*.*03 | *−*0*.*57 | 0*.*82 | – | *−*0*.*04 | *−*0*.*49 | 0*.*65 | – | *−*0*.*01 | *−*1*.*38 | 0*.*51 | – |

The columns of this table presents the following information. **Col.1**: variable of interest; **Col.2**: indicates if the variable is reverse, that is multiplied by -1; **Col.3**: estimated treatment effect for nonmigrants; **Col.4**: Hedges g effect size for non-migrants. **Col.5**: the single hypothesis one-sided mid-p-value based on 15.000 permutations draws. Test statistic uses the pre-pivoted treatment effect estimate and the permutation scheme is either a na¨ıve or block permutation. **Col.6**: the multiple hypothesis testing (stepdown) for *p*-values for non-migrants. **Col.7**: treatment effect for males; **Col.8**: effect size for males; **Col.9**: mid-p-value for males; **Col.10**: stepdown *p*-values for males. **Col.11**: treatment effect for females; **Col.12**: effect size for females; **Col.13**: mid-p-value for females; **Col.14**: stepdown *p*-values for females. Estimates are based on a block permutation inference conditional on main variables at the onset of the intervention.

### Table S8. Comparison vs. No-treatment Conditional Inference on Cognitive, Psychosocial and Personality Outcomes

21

|  | **No-treatment (n=47)** | **Non-stunted (n=64)** | **Comparison** | **Effect** | **Confidence** | **Asymptotic** (two-sided) | **Permutation** (two-sided) |
| --- | --- | --- | --- | --- | --- | --- | --- |
|  | **Mean Std.Dev.** | **Mean Std.Dev.** | **Effects** | **Size** | **Interval** | *t***-stat Single** *p***-val** | **Single** *p***-val Stepdown** |

*WASI IQ Scores*

| Full Scale IQ Score | 71*.*29 | 10*.*91 | 84*.*07 | 12*.*79 | 12*.*78 | 1*.*17 | ( 0*.*73, 1*.*60) | 4*.*03 | **0***.***000** | **0***.***000** | **0***.***000** |
| --- | --- | --- | --- | --- | --- | --- | --- | --- | --- | --- | --- |
| Perceptual Reasoning | 70*.*28 | 11*.*74 | 84*.*26 | 12*.*95 | 13*.*98 | 1*.*19 | ( 0*.*76, 1*.*62) | 4*.*25 | **0***.***000** | **0***.***000** | **0***.***000** |
| Verbal Composite Score | 76*.*95 | 12*.*58 | 86*.*96 | 13*.*78 | 10*.*01 | 0*.*79 | ( 0*.*35, 1*.*23) | 2*.*86 | **0***.***005** | **0***.***001** | **0***.***001** |
| Rank Mean | 0*.*34 | 0*.*21 | 0*.*61 | 0*.*27 | 0*.*27 | 1*.*29 | ( 0*.*93, 1*.*66) | 4*.*23 | **0***.***000** | **0***.***000** | – |
| *Executive Function* |  |  |  |  |  |  |  |  |  |  |  |
| Fluency: Switching Accuracy | 8*.*23 | 2*.*91 | 9*.*13 | 2*.*74 | 0*.*90 | 0*.*31 | ( *−*0*.*11, 0*.*75) | 1*.*22 | 0*.*226 | 0*.*159 | 0*.*254 |
| Fluency: % Switching Accuracy | 8*.*66 | 3*.*41 | 9*.*41 | 2*.*58 | 0*.*75 | 0*.*22 | ( *−*0*.*17, 0*.*67) | 0*.*96 | 0*.*340 | 0*.*298 | 0*.*298 |
| Card Sorting Total Score | 4*.*19 | 2*.*78 | 7*.*03 | 3*.*41 | 2*.*84 | 1*.*02 | ( 0*.*58, 1*.*45) | 3*.*41 | **0***.***001** | **0***.***000** | **0***.***000** |
| Card Sort Description | 4*.*25 | 2*.*64 | 7*.*06 | 3*.*38 | 2*.*81 | 1*.*06 | ( 0*.*61, 1*.*48) | 3*.*44 | **0***.***001** | **0***.***000** | **0***.***000** |
| Tower Achievement Score | 8*.*42 | 3*.*27 | 9*.*43 | 2*.*60 | 1*.*01 | 0*.*31 | ( *−*0*.*11, 0*.*74) | 1*.*32 | 0*.*189 | 0*.*152 | 0*.*340 |
| Rank Mean | 0*.*42 | 0*.*19 | 0*.*56 | 0*.*19 | 0*.*14 | 0*.*72 | ( 0*.*36, 1*.*07) | 2*.*72 | **0***.***008** | **0***.***001** | – |
| *Mental Health* |  |  |  |  |  |  |  |  |  |  |  |
| Depressive symptoms | *−*20*.*62 | 9*.*99 | *−*17*.*36 | 10*.*02 | 3*.*26 | 0*.*33 | ( *−*0*.*10, 0*.*78) | 1*.*23 | 0*.*221 | 0*.*148 | 0*.*238 |
| Anxiety Total Score | *−*42*.*86 | 8*.*48 | *−*42*.*24 | 9*.*36 | 0*.*62 | 0*.*07 | ( *−*0*.*38, 0*.*52) | 0*.*26 | 0*.*794 | 0*.*744 | 0*.*744 |
| Social inhibition | *−*6*.*77 | 3*.*20 | *−*4*.*96 | 3*.*57 | 1*.*81 | 0*.*56 | ( 0*.*13, 1*.*00) | 2*.*00 | **0***.***048** | **0***.***012** | **0***.***032** |
| Rank Mean | 0*.*45 | 0*.*22 | 0*.*54 | 0*.*21 | 0*.*09 | 0*.*42 | ( 0*.*06, 0*.*78) | 1*.*63 | 0*.*105 | **0***.***058** | – |
| *Psychosocial Skills* |  |  |  |  |  |  |  |  |  |  |  |
| Self esteem - Rosenberg Score | 21*.*69 | 5*.*36 | 22*.*08 | 4*.*85 | 0*.*39 | 0*.*07 | ( *−*0*.*35, 0*.*51) | 0*.*29 | 0*.*771 | 0*.*737 | 0*.*737 |
| Grit score | 24*.*88 | 4*.*59 | 25*.*26 | 4*.*03 | 0*.*38 | 0*.*08 | ( *−*0*.*35, 0*.*54) | 0*.*34 | 0*.*736 | 0*.*712 | 0*.*902 |
| Self-control Measure | 8*.*91 | 3*.*23 | 9*.*72 | 2*.*81 | 0*.*81 | 0*.*25 | ( *−*0*.*18, 0*.*68) | 1*.*03 | 0*.*306 | 0*.*251 | 0*.*542 |
| Rank Mean  *Personality Traits* | 0*.*48 | 0*.*23 | 0*.*52 | 0*.*21 | 0*.*04 | 0*.*19 | ( *−*0*.*18, 0*.*55) | 0*.*74 | 0*.*461 | 0*.*398 | – |
| Extraversion | 8*.*38 | 2*.*38 | 8*.*71 | 2*.*71 | 0*.*33 | 0*.*14 | ( *−*0*.*29, 0*.*57) | 0*.*49 | 0*.*625 | 0*.*524 | 0*.*524 |
| Agreeableness | 11*.*89 | 2*.*34 | 11*.*29 | 2*.*25 | *−*0*.*60 | *−*0*.*26 | ( *−*0*.*67, 0*.*19) | *−*1*.*00 | 0*.*321 | 0*.*232 | 0*.*535 |
| Conscientious | 11*.*28 | 2*.*69 | 12*.*25 | 2*.*26 | 0*.*97 | 0*.*36 | ( *−*0*.*02, 0*.*84) | 1*.*49 | 0*.*139 | **0***.***097** | 0*.*387 |
| Emotional Stability | 10*.*17 | 2*.*75 | 9*.*63 | 3*.*07 | *−*0*.*54 | *−*0*.*20 | ( *−*0*.*62, 0*.*23) | *−*0*.*70 | 0*.*487 | 0*.*361 | 0*.*593 |
| Open to Experiences | 10*.*96 | 2*.*36 | 10*.*20 | 2*.*46 | *−*0*.*75 | *−*0*.*32 | ( *−*0*.*73, 0*.*14) | *−*1*.*18 | 0*.*242 | 0*.*146 | 0*.*456 |
| Rank Mean | 0*.*50 | 0*.*15 | 0*.*50 | 0*.*16 | *−*0*.*01 | *−*0*.*05 | ( *−*0*.*42, 0*.*31) | *−*0*.*17 | 0*.*863 | 0*.*834 | – |

The columns of this table presents the following information. **Col.1**: Variable of interest; **Col.2**: No-treatment group mean; **Col.3**: Standard deviation; **Col.4**: Comparison group mean; **Col.5**: Standard deviation; **Col.6**: Estimated difference in means between participants; **Col.7**: Hedges g effect size according to Rosenthal and Rosnow (1991) and Becker (2000). **Col.8**: 95% confidence interval for the effect size. **Col.9**: t-statistic associated with the treatment effect. **Col.10**: Asymptotic two-sided *p*-value for the single hypothesis testing of no difference between non-stunted outcome mean versus no-treatment mean. **Col.11**: Single hypothesis two-sided mid-p-value based on 15.000 permutations draws. Test statistic uses the pre-pivoted effect size estimate and a block permutation scheme. **Col.12**: Multiple hypothesis testing (stepdown) for *p*-values in column 10. Estimates are based on a block permutation inference conditional on main variables at the onset of the intervention.

22

### Table S9. Comparison vs. No-treatment Conditional Inference on Risk Taking and Violence Behaviors

|  | **No-treatment (n=47)** | **Non-stunted (n=64)** | **Comparison** | **Effect** | **Confidence** | **Asymptotic** (two-sided) | **Permutation** (two-sided) |
| --- | --- | --- | --- | --- | --- | --- | --- |
|  | **Mean Std.Dev.** | **Mean Std.Dev.** | **Effects** | **Size** | **Interval** | *t***-stat Single** *p***-val** | **Single** *p***-val Stepdown** |

*Substance Abuse (WHO)*

| Aggregate Alcohol Score | *−*3*.*48 | 2*.*82 | *−*3*.*31 | 2*.*73 | 0*.*17 | 0*.*06 | ( *−*0*.*34, 0*.*54) | 0*.*23 | 0*.*818 | 0*.*782 | 0*.*782 |
| --- | --- | --- | --- | --- | --- | --- | --- | --- | --- | --- | --- |
| Aggregate Ganja Score (WHO) | *−*3*.*14 | 3*.*92 | *−*2*.*35 | 3*.*86 | 0*.*79 | 0*.*20 | ( *−*0*.*21, 0*.*70) | 0*.*77 | 0*.*441 | 0*.*373 | 0*.*581 |
| Rank Mean  *Risks Taking Factor Scores* | 0*.*48 | 0*.*20 | 0*.*51 | 0*.*19 | 0*.*03 | 0*.*17 | ( *−*0*.*20, 0*.*53) | 0*.*68 | 0*.*501 | 0*.*431 | – |
| General Risk & Finance | *−*3*.*74 | 0*.*86 | *−*3*.*79 | 1*.*04 | *−*0*.*05 | *−*0*.*06 | ( *−*0*.*55, 0*.*36) | *−*0*.*20 | 0*.*840 | 0*.*802 | 0*.*802 |
| Health, Work & Trust | *−*3*.*30 | 0*.*93 | *−*2*.*76 | 0*.*92 | 0*.*54 | 0*.*58 | ( 0*.*15, 1*.*02) | 2*.*23 | **0***.***028** | **0***.***009** | **0***.***018** |
| Rank Mean | 0*.*46 | 0*.*19 | 0*.*53 | 0*.*23 | 0*.*06 | 0*.*32 | ( *−*0*.*04, 0*.*70) | 1*.*09 | 0*.*276 | 0*.*146 | – |
| *Violence Factor Scores* |  |  |  |  |  |  |  |  |  |  |  |
| Factor Score Fights & Weapons | *−*0*.*05 | 1*.*05 | *−*0*.*10 | 1*.*20 | *−*0*.*06 | *−*0*.*05 | ( *−*0*.*42, 0*.*51) | *−*0*.*18 | 0*.*854 | 0*.*809 | 0*.*809 |
| Factor Score Guns & Gangs | 0*.*40 | 0*.*58 | *−*0*.*43 | 1*.*21 | *−*0*.*83 | *−*1*.*40 | ( *−*1*.*77, *−*0*.*79) | *−*3*.*17 | **0***.***002** | **0***.***000** | **0***.***001** |
| Rank Mean | 0*.*54 | 0*.*06 | 0*.*47 | 0*.*16 | *−*0*.*07 | *−*1*.*21 | ( *−*1*.*69, *−*0*.*86) | *−*2*.*14 | **0***.***035** | **0***.***001** | – |

The columns of this table presents the following information. **Col.1**: Variable of interest; **Col.2**: No-treatment mean; **Col.3**: Standard deviation; **Col.4**: Comparison group mean; **Col.5**: Standard deviation; **Col.6**: Estimated difference in means between participants; **Col.7**: Hedges g effect size according to Rosenthal and Rosnow (1991) and Becker (2000). **Col.8**: 95% confidence interval for the effect size. **Col.9**: t-statistic associated with the treatment effect. **Col.10**: Asymptotic two-sided *p*-value for the single hypothesis testing of no difference between non-stunted mean versus no-treatment mean. **Col.11**: Single hypothesis two-sided mid-p-value based on 15.000 permutations draws. Test statistic uses the pre-pivoted effect size estimate and a block permutation scheme. **Col.12**: Multiple hypothesis testing (stepdown) for *p*-values in column 10. Estimates are based on a block permutation inference conditional on main variables at the onset of the intervention.

### Table S10. Comparison vs. Treatment Conditional Inference on Cognitive, Psychosocial and Personality Outcomes

23

|  | **Treated (n=48)** | **Non-stunted (n=64)** | **Non-stunted** | **Effect** | **Confidence** | **Asymptotic** (two-sided) | **Permutation** (two-sided) |
| --- | --- | --- | --- | --- | --- | --- | --- |
|  | **Mean Std.Dev.** | **Mean Std.Dev.** | **Effects** | **Size** | **Interval** | *t***-stat Single** *p***-val** | **Single** *p***-val Stepdown** |

*WASI IQ Scores*

| Full Scale IQ Score | 76*.*35 | 11*.*61 | 83*.*55 | 12*.*81 | 7*.*20 | 0*.*62 | ( 0*.*18, 1*.*07) | 2*.*21 | **0***.***029** | **0***.***007** | **0***.***013** |
| --- | --- | --- | --- | --- | --- | --- | --- | --- | --- | --- | --- |
| Perceptual Reasoning | 76*.*02 | 12*.*69 | 83*.*89 | 13*.*05 | 7*.*87 | 0*.*62 | ( 0*.*19, 1*.*08) | 2*.*30 | **0***.***023** | **0***.***007** | **0***.***012** |
| Verbal Composite Score | 80*.*66 | 12*.*20 | 86*.*35 | 13*.*70 | 5*.*69 | 0*.*47 | ( 0*.*03, 0*.*92) | 1*.*64 | 0*.*104 | **0***.***040** | **0***.***040** |
| Rank Mean | 0*.*41 | 0*.*25 | 0*.*57 | 0*.*27 | 0*.*17 | 0*.*66 | ( 0*.*29, 1*.*03) | 2*.*40 | **0***.***018** | **0***.***004** | – |
| *Executive Function* |  |  |  |  |  |  |  |  |  |  |  |
| Fluency: Switching Accuracy | 8*.*74 | 3*.*15 | 9*.*05 | 2*.*75 | 0*.*31 | 0*.*10 | ( *−*0*.*33, 0*.*54) | 0*.*40 | 0*.*690 | 0*.*653 | 0*.*951 |
| Fluency: % Switching Accuracy | 9*.*60 | 2*.*96 | 9*.*38 | 2*.*62 | *−*0*.*22 | *−*0*.*07 | ( *−*0*.*48, 0*.*39) | *−*0*.*30 | 0*.*766 | 0*.*737 | 0*.*737 |
| Card Sorting Total Score | 5*.*71 | 2*.*98 | 6*.*94 | 3*.*38 | 1*.*23 | 0*.*41 | ( *−*0*.*04, 0*.*88) | 1*.*45 | 0*.*151 | **0***.***074** | 0*.*244 |
| Card Sort Description | 5*.*54 | 3*.*01 | 7*.*00 | 3*.*34 | 1*.*45 | 0*.*48 | ( 0*.*03, 0*.*93) | 1*.*71 | **0***.***090** | **0***.***036** | 0*.*131 |
| Tower Achievement Score | 9*.*18 | 2*.*15 | 9*.*38 | 2*.*60 | 0*.*20 | 0*.*09 | ( *−*0*.*36, 0*.*55) | 0*.*31 | 0*.*761 | 0*.*691 | 0*.*904 |
| Rank Mean | 0*.*47 | 0*.*19 | 0*.*52 | 0*.*20 | 0*.*05 | 0*.*27 | ( *−*0*.*11, 0*.*64) | 1*.*00 | 0*.*321 | 0*.*227 | – |
| *Mental Health* |  |  |  |  |  |  |  |  |  |  |  |
| Depressive symptoms | *−*15*.*60 | 9*.*85 | *−*17*.*47 | 10*.*15 | *−*1*.*86 | *−*0*.*19 | ( *−*0*.*61, 0*.*28) | *−*0*.*70 | 0*.*485 | 0*.*408 | 0*.*651 |
| Anxiety Total Score | *−*40*.*64 | 6*.*99 | *−*42*.*24 | 9*.*40 | *−*1*.*60 | *−*0*.*23 | ( *−*0*.*70, 0*.*25) | *−*0*.*71 | 0*.*478 | 0*.*341 | 0*.*682 |
| Social inhibition | *−*5*.*67 | 3*.*54 | *−*5*.*07 | 3*.*57 | 0*.*60 | 0*.*17 | ( *−*0*.*23, 0*.*62) | 0*.*63 | 0*.*527 | 0*.*435 | 0*.*435 |
| Rank Mean  *Psychosocial Skills* | 0*.*51 | 0*.*20 | 0*.*49 | 0*.*21 | *−*0*.*02 | *−*0*.*11 | ( *−*0*.*48, 0*.*27) | *−*0*.*39 | 0*.*698 | 0*.*636 | – |
| Self esteem - Rosenberg Score | 22*.*64 | 4*.*44 | 22*.*10 | 4*.*90 | *−*0*.*54 | *−*0*.*12 | ( *−*0*.*56, 0*.*35) | *−*0*.*43 | 0*.*665 | 0*.*588 | 0*.*824 |
| Grit score | 26*.*95 | 3*.*78 | 25*.*21 | 4*.*03 | *−*1*.*74 | *−*0*.*46 | ( *−*0*.*93, 0*.*00) | *−*1*.*68 | **0***.***097** | **0***.***053** | 0*.*143 |
| Self-control Measure | 9*.*74 | 2*.*97 | 9*.*60 | 2*.*82 | *−*0*.*14 | *−*0*.*05 | ( *−*0*.*49, 0*.*41) | *−*0*.*18 | 0*.*859 | 0*.*843 | 0*.*843 |
| Rank Mean  *Personality Traits* | 0*.*53 | 0*.*21 | 0*.*47 | 0*.*22 | *−*0*.*06 | *−*0*.*29 | ( *−*0*.*67, 0*.*10) | *−*1*.*06 | 0*.*293 | 0*.*218 | – |
| Extraversion | 8*.*41 | 2*.*76 | 8*.*63 | 2*.*71 | 0*.*21 | 0*.*08 | ( *−*0*.*36, 0*.*53) | 0*.*29 | 0*.*769 | 0*.*731 | 0*.*731 |
| Agreeableness | 11*.*88 | 2*.*41 | 11*.*26 | 2*.*22 | *−*0*.*61 | *−*0*.*25 | ( *−*0*.*68, 0*.*19) | *−*1*.*01 | 0*.*317 | 0*.*251 | 0*.*438 |
| Conscientious | 13*.*07 | 1*.*84 | 12*.*23 | 2*.*27 | *−*0*.*84 | *−*0*.*46 | ( *−*0*.*83, 0*.*12) | *−*1*.*51 | 0*.*134 | **0***.***058** | 0*.*246 |
| Emotional Stability | 10*.*58 | 2*.*47 | 9*.*60 | 3*.*08 | *−*0*.*98 | *−*0*.*40 | ( *−*0*.*83, 0*.*05) | *−*1*.*31 | 0*.*194 | **0***.***075** | 0*.*259 |
| Open to Experiences | 11*.*03 | 2*.*73 | 10*.*15 | 2*.*49 | *−*0*.*89 | *−*0*.*33 | ( *−*0*.*75, 0*.*14) | *−*1*.*29 | 0*.*200 | 0*.*143 | 0*.*368 |
| Rank Mean | 0*.*54 | 0*.*15 | 0*.*47 | 0*.*15 | *−*0*.*07 | *−*0*.*47 | ( *−*0*.*85, *−*0*.*10) | *−*1*.*76 | **0***.***081** | **0***.***040** | – |

The columns of this table presents the following information. **Col.1**: Variable of interest; **Col.2**: Treatment group mean; **Col.3**: Standard deviation; **Col.4**: Comparison group mean; **Col.5**: Standard deviation; **Col.6**: Estimated difference in means between participants; **Col.7**: Hedges g effect size according to Rosenthal and Rosnow (1991) and Becker (2000). **Col.8**: 95% confidence interval for the effect size. **Col.9**: t-statistic associated with the treatment effect. **Col.10**: Asymptotic two-sided *p*-value for the single hypothesis testing of no difference between non-stunted versus treated. **Col.11**: Single hypothesis two-sided mid-p-value based on 15.000 permutations draws. Test statistic uses the pre-pivoted effect size estimate and a block permutation scheme. **Col.12**: Multiple hypothesis testing (stepdown) for *p*-values in column 10. Estimates are based on a block permutation inference conditional on main variables at the onset of the intervention.

24

### Table S11. Comparison vs. Treatment Conditional Inference on Risk Taking and Violence Behaviors

|  | **Treated (n=48)** | **Non-stunted (n=64)** | **Non-stunted** | **Effect** | **Confidence** | **Asymptotic** (two-sided) | **Permutation** (two-sided) |
| --- | --- | --- | --- | --- | --- | --- | --- |
|  | **Mean Std.Dev.** | **Mean Std.Dev.** | **Effects** | **Size** | **Interval** | *t***-stat Single** *p***-val** | **Single** *p***-val Stepdown** |

*Substance Abuse (WHO)*

| Aggregate Alcohol Score | *−*2*.*58 | 3*.*23 | *−*3*.*41 | 2*.*75 | *−*0*.*82 | *−*0*.*26 | ( *−*0*.*63, 0*.*28) | *−*1*.*05 | 0*.*296 | 0*.*240 | 0*.*408 |
| --- | --- | --- | --- | --- | --- | --- | --- | --- | --- | --- | --- |
| Aggregate Ganja Score (WHO) | *−*2*.*27 | 3*.*71 | *−*2*.*53 | 3*.*88 | *−*0*.*27 | *−*0*.*07 | ( *−*0*.*49, 0*.*43) | *−*0*.*26 | 0*.*793 | 0*.*748 | 0*.*748 |
| Rank Mean  *Risks Taking Factor Scores* | 0*.*53 | 0*.*21 | 0*.*48 | 0*.*18 | *−*0*.*04 | *−*0*.*21 | ( *−*0*.*58, 0*.*16) | *−*0*.*84 | 0*.*401 | 0*.*359 | – |
| General Risk & Finance | *−*3*.*51 | 1*.*08 | *−*3*.*82 | 1*.*03 | *−*0*.*31 | *−*0*.*29 | ( *−*0*.*78, 0*.*15) | *−*1*.*11 | 0*.*269 | 0*.*222 | 0*.*377 |
| Health, Work & Trust | *−*2*.*78 | 1*.*07 | *−*2*.*73 | 0*.*91 | 0*.*06 | 0*.*05 | ( *−*0*.*38, 0*.*49) | 0*.*22 | 0*.*826 | 0*.*809 | 0*.*809 |
| Rank Mean  *Violence Factor Scores* | 0*.*52 | 0*.*24 | 0*.*49 | 0*.*22 | *−*0*.*03 | *−*0*.*13 | ( *−*0*.*49, 0*.*25) | *−*0*.*52 | 0*.*605 | 0*.*563 | – |
| Factor Score Fights & Weapons | 0*.*19 | 0*.*51 | *−*0*.*11 | 1*.*19 | *−*0*.*29 | *−*0*.*56 | ( *−*0*.*96, 0*.*10) | *−*1*.*14 | 0*.*257 | **0***.***043** | **0***.***043** |
| Factor Score Guns & Gangs | 0*.*34 | 0*.*64 | *−*0*.*47 | 1*.*21 | *−*0*.*81 | *−*1*.*27 | ( *−*1*.*65, *−*0*.*68) | *−*3*.*03 | **0***.***003** | **0***.***001** | **0***.***002** |
| Rank Mean | 0*.*54 | 0*.*12 | 0*.*47 | 0*.*16 | *−*0*.*06 | *−*0*.*56 | ( *−*0*.*99, *−*0*.*22) | *−*1*.*67 | **0***.***098** | **0***.***024** | – |

The columns of this table presents the following information. **Col.1**: Variable of interest; **Col.2**: Treatment group mean; **Col.3**: Standard deviation; **Col.4**: Comparison group mean; **Col.5**: Standard deviation; **Col.6**: Estimated difference in means between participants; **Col.7**: Hedges g effect size according to Rosenthal and Rosnow (1991) and Becker (2000). **Col.8**: 95% confidence interval for the effect size. **Col.9**: t-statistic associated with the treatment effect. **Col.10**: Asymptotic two-sided *p*-value for the single hypothesis testing of no difference between non-stunted versus treated. **Col.11**: Single hypothesis two-sided mid-p-value based on 15.000 permutations draws. Test statistic uses the pre-pivoted effect size estimate and a block permutation scheme. **Col.12**: Multiple hypothesis testing (stepdown) for *p*-values in column 10. Estimates are based on a block permutation inference conditional on main variables at the onset of the intervention.

**References**

Campbell, F., G. Conti, J. J. Heckman, S. H. Moon, R. Pinto, E. Pungello, and Y. Pan (2013).

Early childhood investments substantially boost adult health. Under review, *Science*.

Gertler, P., J. J. Heckman, R. Pinto, A. Zanolini, C. Vermeersch, S. Walker, S. Chang, and S. M. Grantham-McGregor (2014). Labor market returns to an early childhood stimulation intervention in Jamaica. *Science 344* (6187), 998–1001.

Glynn, A. N. and K. M. Quinn (2010a). An introduction to the augmented inverse propensity weighted estimator. *Political Analysis 18* (1), 36–56.

Glynn, A. N. and K. M. Quinn (2010b). An introduction to the augmented inverse propensity weighted estimator. *Journal of the American Statistical Association* (18), 36–56.

Heckman, J. J., S. H. Moon, R. Pinto, P. A. Savelyev, and A. Q. Yavitz (2010, July). Analyzing social experiments as implemented: A reexamination of the evidence from the HighScope Perry Preschool Program. *Quantitative Economics 1* (1), 1–46.

Robins, J. M. (1999). Robust estimation in sequentially ignorable missing data and causal infer- ence models. *Proceedings of the American Statistical Association Section on Bayesian Statistical Science*, 6–10.

Robins, J. M., A. Rotnitzky, and L. P. Zhao (1994). Estimation of regression coefficients when some regressors are not always observed. *Journal of the American Statistical Association 89* (427), 846– 866.

Romano, J. P. and M. Wolf (2005). Stepwise multiple testing as formalized data snooping. *Econo- metrica 73* (4), 1237–1282.

Rosnow, R. L. and R. Rosenthal (2003). Effect sizes for experimenting psychologists. *Canadian Journal of Experimental Psychology* (57), 221–237.

Scharfstein, D. O., A. Rotnitzky, and J. M. Robins (1999). Adjusting for nonignorable drop-out us- ing semiparametric nonresponse models. *Journal of the American Statistical Association 94* (448), 1096–1120.

25
